# LincRNA-EPS alleviates osteoclastogenesis under inflammatory microenvironment through preventing excessive iron metabolism

**DOI:** 10.1038/s41419-026-08716-y

**Published:** 2026-04-03

**Authors:** Jin Wang, Yabing Wang, Zhanwei Zhang, Xin Wang, Jiansheng Su

**Affiliations:** 1 Shanghai Engineering Research Center of Tooth Restoration and Regeneration & Tongji Research Institute of Stomatology & Department of Prosthodontics, Shanghai Tongji Stomatological Hospital and Dental School, Tongji University, Shanghai, China; 2 Shanghai Engineering Research Center of Tooth Restoration and Regeneration & Tongji Research Institute of Stomatology & Department of Endodontics, Shanghai Tongji Stomatological Hospital and Dental School, Tongji University, Shanghai, China

**Keywords:** Cell biology, Long non-coding RNAs, Osteoimmunology

## Abstract

The precise regulation of bone homeostasis and the balance between bone resorption and formation in periodontitis remain unclear. This study explores the role of long intergenic noncoding RNA-erythroid prosurvival (lincRNA-EPS) in inflammatory osteoclastogenesis and bone resorption. LincRNA-EPS knockout (KO) worsened LPS-induced alveolar bone resorption in vivo and osteoclast differentiation in vitro. Transcriptomics and protein sequencing showed dysregulated osteoclastogenesis and iron homeostasis without lincRNA-EPS, marked by increased expression of *Lcn2*. Knockdown of Lcn2 in osteoclast precursors (OCPs) resulted in a reduction in the level of iron metabolism and osteoclastogenesis; however, the regulatory response was delayed in KO cells. Correspondingly, overexpression of lincRNA-EPS accelerated the regulation of iron metabolism. Further, reducing Lcn2 levels in wildtype mice alleviated periodontitis-related bone loss, but not in KO mice. Taken together, we identified the critical role of lincRNA-EPS in regulating osteoclastogenesis under inflammatory environment, by preventing excessive iron metabolism caused by Lcn2.

## Introduction

Periodontitis is a chronic inflammatory disease that occurs in the supporting tissues of the teeth, including the gingiva, periodontal ligament and alveolar bone [1]. Alveolar bone resorption, the result of osteoclast activation, is one of the primary clinical manifestations of periodontitis and a major cause of tooth loss, significantly impacting patients’ quality of life [2]. Mechanistically, the pathologic bacteria trigger inflammatory response in periodontal microenvironment, which further stimulates the production of various pro-inflammatory cytokines and Receptor Activator of Nuclear Factor-κB Ligand (RANKL), inducing osteoclast differentiation and activation. Additionally, pathogenic factors such as periodontal bacteria and mechanical stress can directly activate osteoclasts, leading to alveolar bone resorption [2, 3]. Thus, achieving precise control of bone homeostasis by modulating the balance between bone resorption and formation under inflammatory conditions – particularly by inhibiting excessive osteoclast activity – constitutes a critical step in controlling alveolar bone loss and treating periodontitis.

Classically, osteoclasts differentiation depends on RANKL, which formed through the fusion of monocyte- or macrophage-derived osteoclast precursors (OCPs) that originate from hematopoietic lineage [4, 5]. However, in recent years, multiple RANKL-independent and inflammation-related osteoclast differentiation pathways have been identified and are increasingly recognized as potentially highly relevant to pathological bone resorption [6]. For instance, direct stimulation of OCPs by bacterial lipopolysaccharide (LPS) activates Toll-like receptor 4 (TLR4), triggering tumor necrosis factor-α (TNF-α) secretion. This cytokine acts in an autocrine/paracrine manner to promote osteoclast precursor differentiating into mature osteoclasts via the NF-κB pathway [7]. Additionally, metabolic reprogramming mediated by transforming growth factor-β (TGF-β)/TNF can independently drive osteoclast differentiation and maturation without RANKL involvement [8]. That is, osteoclast can be over-activated under inflammatory environment through mechanisms beyond the effect of RANKL, which are essential in inflammatory bone resorption. However, how to suppress the reaction of osteoclast lineage to inflammatory factors remains unknown.

Long intergenic noncoding RNA-erythroid prosurvival (lincRNA-EPS) is capable of controlling nucleosome positioning and suppressing immune response gene transcription [9]. By binding to proteins such as SRSF3 (serine/arginine-rich splicing factor 3) and TDP43 (TAR DNA-binding protein-43), it inhibits the activation of the NF-κB pathway in condylar chondrocytes and gingival fibroblasts, respectively, thereby reducing inflammatory responses in tissues and mitigating bone damage in temporomandibular joint osteoarthritis and periodontitis [10, 11]. Overall, lincRNA-EPS can suppress the inflammatory response in cells [12, 13]. Since inflammatory factors increased osteoclast differentiation, lincRNA-EPS may also work in the process of osteoclastogenesis under inflammatory conditions. Our former research found that overexpression of lincRNA-EPS inhibits LPS-induced osteoclastogenesis in cell line Raw264.7 [14]. However, its direct regulatory effect and specific mechanisms on osteoclast differentiation in an inflammatory environment remain unknown. Therefore, exploring whether and by what mechanisms lincRNA‑EPS regulates inflammation‑driven osteoclastogenesis may not only reveal a novel hub linking inflammatory responses to abnormal bone resorption, but also provide crucial theoretical insights and potential targets for modulating the osteoimmune microenvironment and developing new therapeutic strategies.

With this goal, we utilized an LPS-induced periodontitis mouse model and an LPS-induced osteoclastogenesis model by bone marrow-derived macrophages (BMDMs). In this article, we discovered that lincRNA-EPS regulated iron metabolism homeostasis in osteoclasts, with the molecule lipocalin-2 (Lcn2) playing a critical role in this process. The deficiency of lincRNA-EPS promoted Lcn2 expression and iron metabolism, with upregulated osteoclast differentiation. This study delineates the regulatory role of lincRNA-EPS in inflammation-driven osteoclastogenesis and reveals its mediation of iron metabolism, which helps understanding pathology and developing targeted therapy of inflammatory bone disorders.

## Results

### LincRNA-EPS knockout exacerbated alveolar bone resorption in vivo and LPS-induced osteoclast differentiation in vitro

**Fig. 1:**
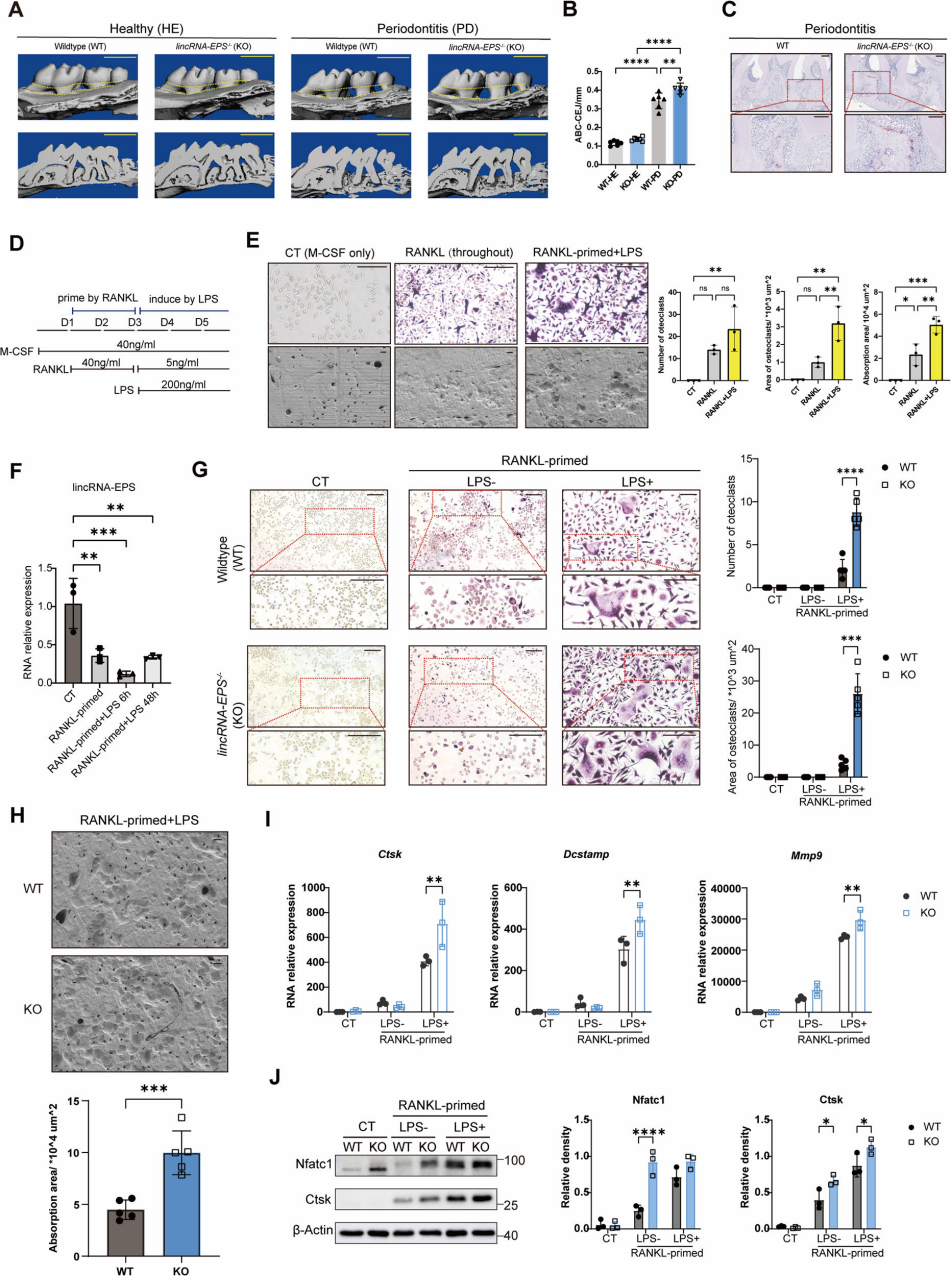
LincRNA-EPS knockout exacerbated alveolar bone resorption in vivo and LPS-induced osteoclast differentiation in vitro. **A** Micro-CT reconstruction of maxillary alveolar bone in wild-type (WT) and *lincRNA-EPS*^-/-^ (KO) mice under healthy and LPS-induced periodontitis conditions. Scale bar: 1 mm. **B** Quantification of the cementoenamel junction to alveolar bone crest (CEJ-ABC) distance (*n* = 6). **C** TRAP staining showing osteoclast activity in periodontitis-affected periodontal tissues. Scale bar: 100 μm. **D** Schematic of the LPS-induced osteoclastogenesis model in vitro using bone marrow-derived macrophages (BMDMs). **E** TRAP staining of osteoclasts (upper) and bone resorption pit morphology shown by electron microscopy (lower) under various induction conditions. Quantification results of osteoclasts and resorption pits are shown on the right (*n* = 3). Scale bar: 20 μm. **F** The expression of lincRNA-EPS during LPS-induced osteoclastogenesis (*n* = 3). **G** TRAP staining of osteoclasts differentiated from WT and KO BMDMs with LPS stimulation after RANKL-primed (*n* = 5). Scale bar: 20 μm. **H** Bone resorption pits of bone slices co-cultured with WT and KO osteoclasts (*n* = 5). Scale bar: 20 μm. **I** qRT-PCR analysis of osteoclastogenesis markers (*Ctsk*, *Dcstamp, Mmp9*) (*n* = 3). The expression levels of genes were normalized to β-Actin levels. **J** Western blot analysis of osteoclastogenesis marker proteins (Nfatc1, Ctsk) (*n* = 3). One-way ANOVA with Tukey-Kramer test was used in **B**, **E**, and **F**. Two-way ANOVA with Tukey-Kramer test was used in **G**, **I** and **J**. Data were presented as mean ± SD. Significance levels were denoted as follows: *P < 0.05, **P < 0.01, ***P < 0.001, ****P < 0.0001, ns refers to no significant difference.

Periodontitis was induced via LPS-saturated silk thread ligation in wildtype (WT) and *lincRNA-EPS*^*-/-*^ (KO) mice. Alveolar bone resorption was evaluated by micro-CT and measured as the distance between alveolar bone crest and cemento-enamel junction (ABC-CEJ). As micro-CT analysis showed, the periodontitis model was successfully established after silk-ligation, and caused more severe alveolar bone resorption in KO mice (Fig. 1A, B). TRAP staining revealed a higher number of osteoclasts surrounding the absorbed alveolar crest in KO mice (Fig. 1C). The effect of lincRNA-EPS in osteoclast differentiation was also investigated in vitro. To mimic the osteoclast differentiation process under inflammatory environment, bone marrow-derived macrophages (BMDMs) isolated from both genotypes were stimulated with macrophage colony-stimulating factor (M-CSF), primed by receptor activator for nuclear factor-κB ligand (RANKL) for two days to form OCPs, and then induced by LPS and low-concentration RANKL (Fig. 1D). Compared with induced only by RANKL, the stimulus of LPS at the late stage significantly enhanced osteoclast differentiation and bone resorption on bone slices (Fig. 1E). On the other hand, as shown in qRT-PCR, the expression of lincRNA-EPS decreased after stimulation with RANKL and LPS (Fig. 1F). Therefore, LPS-induced osteoclasts differentiated from WT and KO OCPs were compared. More and larger osteoclasts were induced when lincRNA-RPS was deleted (Fig. 1G). Besides, an increase in bone resorption pits was observed on the bone slices co-cultured with KO osteoclasts, indicating a higher bone resorption activity of the knockout cells (Fig. 1H). Meanwhile, the expression of osteoclast marker genes (*Dcstamp, Ctsk, Mmp9*) and representative proteins (Nfatc1, Ctsk) were upregulated in KO osteoclasts (Fig. 1I, J). These data indicates that lincRNA-EPS deletion exacerbate LPS-induced osteoclast differentiation and function both in vivo and in vitro.

### Transcriptomic profiling revealed dysregulated osteoclastogenesis and iron homeostasis in lincRNA-EPS-deficient osteoclasts, represented by up-regulated gene *Lcn2*

**Fig. 2:**
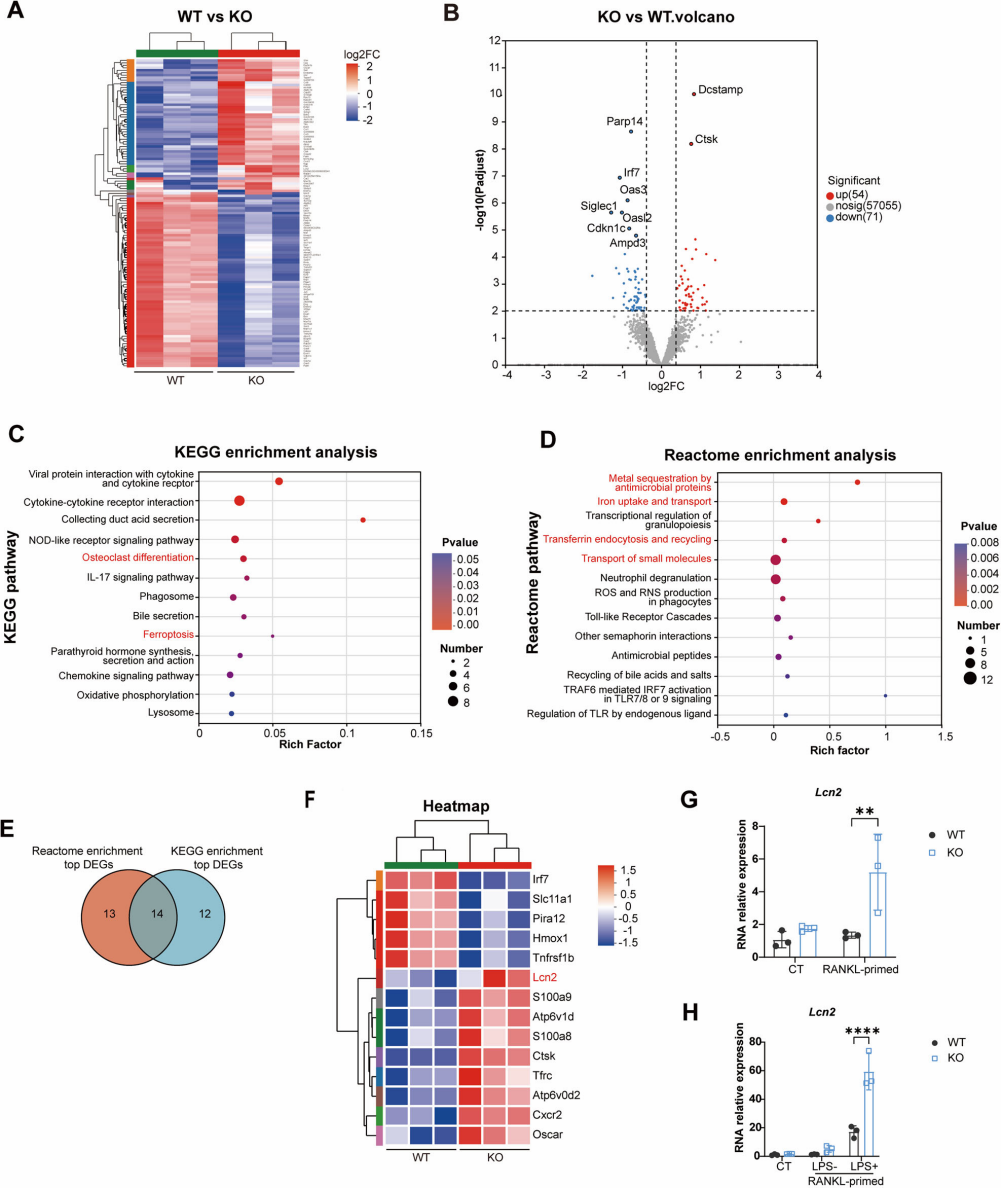
Transcriptomic profiling revealed dysregulated osteoclastogenesis and iron homeostasis in lincRNA-EPS-deficient osteoclasts, represented by up-regulated gene *Lcn2.* **A** Heatmap of significantly differently expressed genes (DEGs) between WT and KO osteoclast precursors ( | log2FC | > 1.3, FDR < 0.01). **B** Volcano plot of DEGs (red: up-regulated in KO; blue: down-regulated in KO). **C** KEGG pathway enrichment of DEGs. **D** Reactome pathway enrichment of DEGs. **E** Venn diagram of overlapped genes from top 10 pathways of KEGG and Reactome enrichment. **F** Heatmap of core dysregulated genes. **G** *Lcn2* mRNA expression after RANKL-primed. (*n* = 3). **H** *Lcn2* mRNA expression after LPS-induced. (*n* = 3). Two-way ANOVA with Tukey-Kramer test was used in **G** and **H**. Data were presented as mean ± SD. **P < 0.01. ****P < 0.0001.

The properties of OCPs greatly affect the generation of osteoclasts [15]. The above findings indicates that KO OCPs are more responsive to LPS stimulation. To investigate the underlying mechanism of lincRNA-EPS affecting osteoclast differentiation, transcriptomics sequencing was performed on WT and KO OCPs which underwent RANKL-priming (Fig. 2A). The top 125 differential expressing genes (DEGs) were used for further analysis. Among them, *Ctsk* and *Dcstamp* were top upregulated genes, indicating increased osteoclastogenesis (Fig.2B). During osteoclast differentiation, extensive metabolic reprogramming occurs, involving changes in oxidative phosphorylation, glycolysis, lipid metabolism, amino acid metabolism [16]. Proper regulation of metabolic activity is crucial for osteoclast differentiation, and metabolic reprogramming can modulate osteoclast function [17]. Therefore, KEGG and Reactome enrichment analyses were performed, and we focused specifically on the alterations in cellular metabolic activity, function, and different signaling pathways between KO and WT OCPs. KEGG pathway enrichment analysis of DEGs showed enrichment in osteoclast differentiation and ferroptosis (Fig. 2C). Besides, pathways related to iron metabolism, including metal sequestration, iron uptake and transport and transferrin endocytosis and recycling, were enriched by Reactome pathway analysis (Fig. 2D). These enrichment results revealed dysregulated iron homeostasis after lincRNA-EPS knockout. Next, to find the key targets of lincRNA-EPS, the DEGs associated with the top 10 pathways from KEGG or Reactome analysis were merged, and Venn analysis was performed (Fig. 2E). As a result, 14 genes were identified and their expression levels were showed in a heatmap (Fig. 2F). Notably, consistent with the Reactome enrichment result, *Lcn2* is one of the most highly up-regulated genes, as well as *Tfrc*, both of which are related to iron transport. The increased expression of *Lcn2* was validated by qRT-PCR (Fig. 2G). Interestingly, the *Lcn2* expression was markedly upregulated by stimulation with LPS, and lincRNA-EPS knockout compounded this change (Fig. 2H). These findings suggest that lincRNA-EPS may regulate osteoclast differentiation through regulation on iron metabolism, and *Lcn2* could be the key regulator gene.

### LincRNA-EPS knockout elevated Lcn2 expression and disrupted iron metabolism

Osteoclast differentiation is a highly energy-consuming biological process. During osteoclastogenesis, iron demand is significantly increased, which plays critical roles in mitochondrial metabolism and reactive oxygen species (ROS) production [18]. Therefore, sufficient and stable cellular iron supply is essential for osteoclast differentiation. On the other hand, Lcn2 is an iron-trafficking protein involved in multiple processes such as apoptosis and innate immunity [19, 20]. It transports iron into or out from cells, depending on its iron-binding form and cell microenvironment [20]. Based on the results in the two previous sections, the difference of *Lcn2* expression of WT and KO cells upon LPS stimulus drives our attention to the function change of mature osteoclast, especially the change of Lcn2 and iron metabolism, therefore, we next focused on Lcn2 and iron metabolism in LPS-induced osteoclastogenesis with or without lincRNA-EPS.

**Fig. 3:**
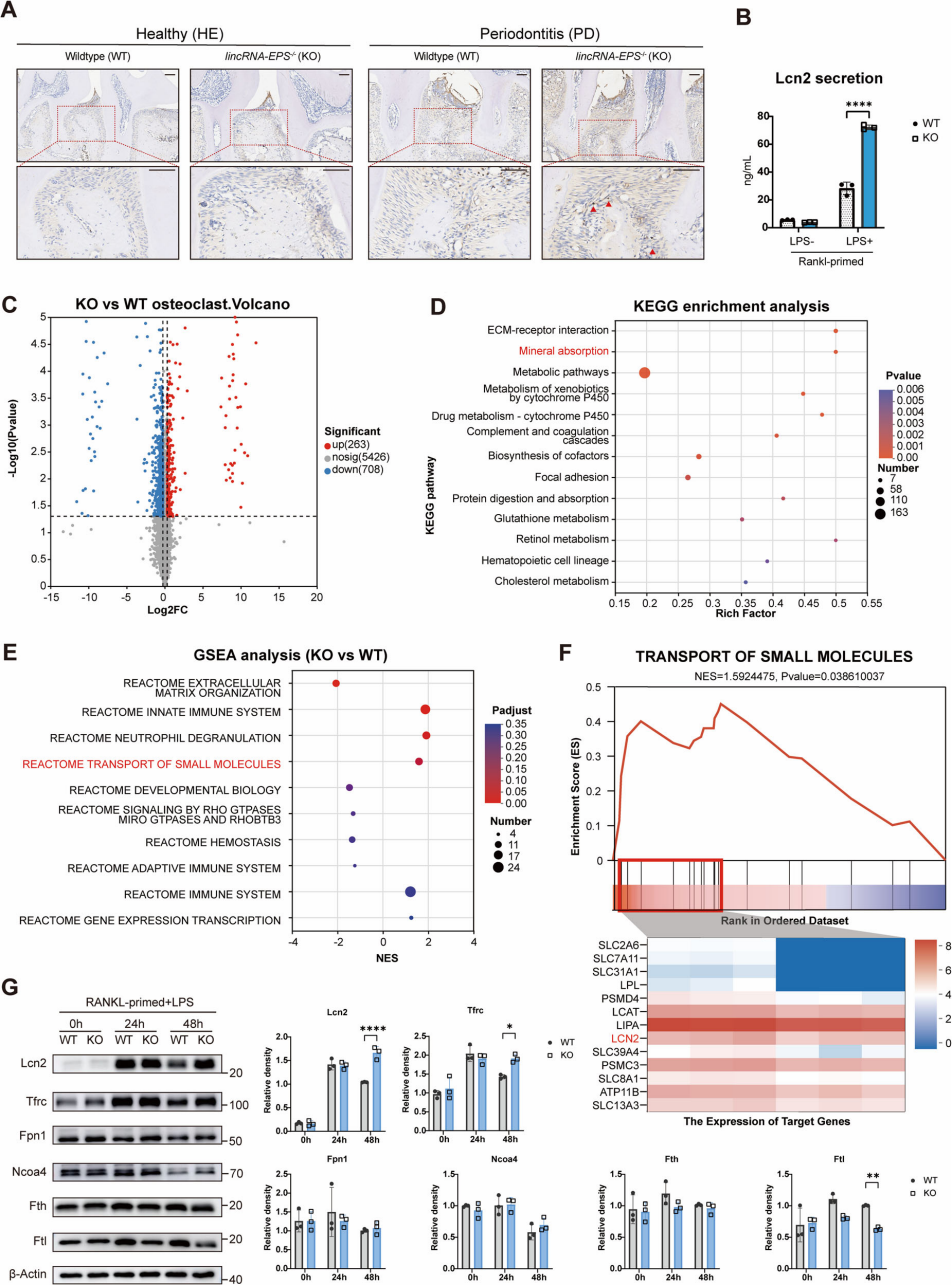
LincRNA-EPS knockout elevated Lcn2 expression and disrupted iron metabolism. **A** Immunohistochemistry (IHC) of Lcn2 in periodontal tissues. Scale bar: 100 μm. **B** ELISA quantification of secreted Lcn2 protein in OCP supernatants (*n* = 3). **C** Volcano plot of differentially expressed proteins in WT vs. KO osteoclasts. **D** KEGG pathway enrichment of differentially expressed proteins. **E** Gene Set Enrichment Analysis (GSEA) of differentially expressed proteins. **F** GSEA of “transport of small molecules” pathway with core enrichment proteins. **G** Western blot analysis of proteins related to iron metabolism during inflammatory osteoclastogenesis. Protein expression levels were quantified by grayscale analysis using ImageJ software (*n* = 3). Two-way ANOVA with Tukey-Kramer test was used in **B** and **G**. Data were presented as mean ± SD. Significance levels were denoted as follows: *P < 0.05, **P < 0.01, ***P < 0.001, ****P < 0.0001.

Firstly, Lcn2 expressions in vivo were detected through immunohistochemical (IHC). The staining results showed higher Lcn2 expression in *lincRNA-EPS*^-/-^ periodontitis tissues (Fig. 3A). In healthy alveolar bone, Lcn2 was primarily localized to the superficial cells of the gingival epithelium, while in periodontitis tissues of wildtype mice, Lcn2 expression extended to the subgingival connective tissue. Moreover, in periodontitis sections of *lincRNA-EPS*^*-/-*^ mice, Lcn2 expression expanded beyond the epithelium and connective tissue, including cells surrounding the alveolar bone (red triangle in Fig. 3A). Furthermore, the Lcn2 secretion level of OCPs upon LPS stimulus was detected through ELISA (Fig. 3B). The results demonstrated that, Lcn2 secretion of OCPs was significantly increased by LPS stimulation, while its secretion was further elevated in *lincRNA-EPS*^*-/-*^ cells under the same LPS stimulation condition.

In order to investigate the changes of osteoclast metabolism and function, proteomics sequencing was performed on wildtype and *lincRNA-EPS*^*-/-*^ osteoclasts (Fig. 3C). KEGG enrichment analysis of differential proteins showed high relation with mineral absorption, which correspond with former results (Fig. 3D). Next, GSEA analysis revealed upregulated function of transport of small molecules (Fig. 3E). The detailed analysis of this protein group (transport of small molecules) listed the representative changed proteins (Fig. 3F). Expectedly, Lcn2 was one of the increased proteins in *lincRNA-EPS*^*-/-*^ osteoclasts. Therefore, the expression of main iron-transporting proteins and related proteins were detected by Western Blotting (Fig. 3G). Lcn2 and Tfrc, which take part in iron transport [20, 21], were upregulated after LPS stimulated (24 h vs 0 h). Notably, their expression maintained at a high level until 48 h in *lincRNA-EPS*^*-/-*^ osteoclasts but not in wildtype cells (48 h). On the other hand, Ncoa4 [22], which plays a critical role in degradation of ferritin, and Fpn1 [23], the iron exporter, were not up-regulated in *lincRNA-EPS*^*-/-*^ cells during osteoclast maturation (0 h and 24 h). The iron storage proteins, Fth and Ftl [24], were maintained and downregulated, respectively (0 h, 24 h and 48 h). These data indicate a sustained and active iron recycling and metabolism in KO osteoclasts.

### *Lcn2* knockdown disturbed iron metabolism and osteoclast maturation in the presence of lincRNA-EPS

**Fig. 4:**
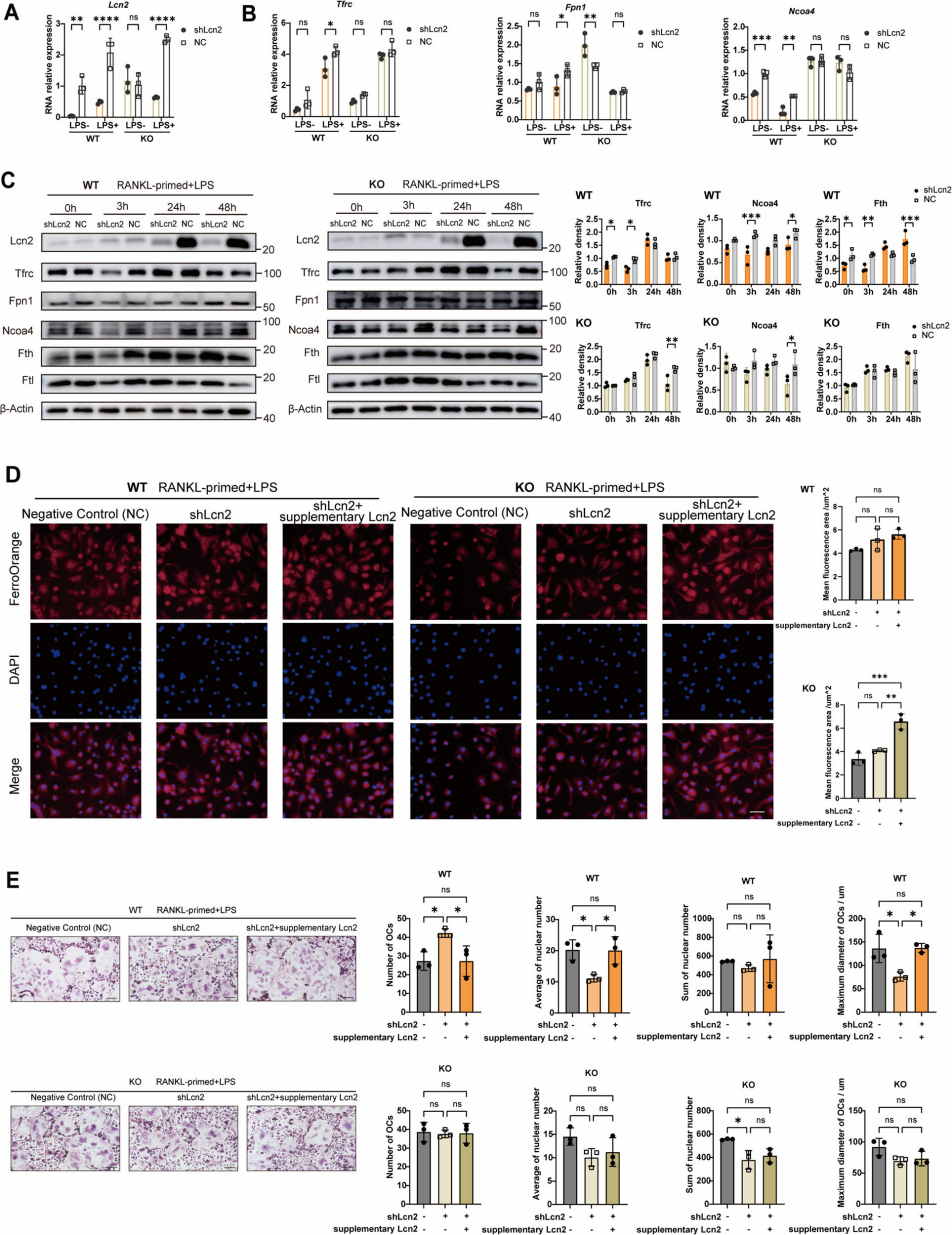
*Lcn2* knockdown disturbed iron metabolism and osteoclast maturation in the presence of lincRNA-EPS. **A** *Lcn2* knockdown efficiency in WT and KO OCPs (*n* = 3). **B** Iron metabolism genes expression of OCPs upon LPS stimulus after *Lcn2* knockdown (*n* = 3). **C** Western blot of iron metabolism proteins in WT and KO cells under RANKL-primed and LPS stimulation after *Lcn2* knockdown (*n* = 3). **D** Fe^2+^ levels in WT and KO cells with *Lcn2* knockdown or recombinant Lcn2 supplementation. Scale bar: 5 μm. Quantification of Fe^2+^ intensity was compared (*n* = 3). **E** TRAP staining showing the osteoclast differentiation after *Lcn2* knockdown and recombinant Lcn2 supplementation. Scale bar: 20um. The number of osteoclasts, the average and sum of nuclei count, and the maximum diameter of osteoclasts were analyzed (*n* = 3). Two-way ANOVA with Tukey-Kramer test was used in **A**–**C**. One-way ANOVA with Tukey–Kramer test was used in **D** and **E**. Data were presented as mean ± SD. Significance levels were denoted as follows: *P < 0.05, **P < 0.01, ***P < 0.001, ****P < 0.0001, ns refers to no significant difference.

To further explore the function of Lcn2 in osteoclast differentiation and the role of lincRNA-EPS in this process, lentivirus was applied to knockdown *Lcn2* expression in WT and KO OCPs, and then LPS were used to induce osteoclastogenesis. Both mRNA and protein expressions of Lcn2 caused by LPS stimulus were significantly reduced in WT and KO osteoclasts (Fig. 4A and 4C). However, the expression patterns of key iron metabolism genes and proteins differed between WT and KO osteoclasts (Fig. 4B, 4C). Among the genes involved in iron supply and storage, *Tfrc*, *Fpn1*, and *Ncoa4* were downregulated following *Lcn2* knockdown in WT osteoclasts, whereas their expression remained unaltered in KO osteoclasts (Fig. 4B, Fig. S1A). Furthermore, in WT osteoclasts, *Lcn2* knockdown decreased the protein expression of Tfrc and Ncoa4 significantly as early as 3 h after LPS stimulation. In contrast, this change in KO osteoclasts was delayed until 48 h (Fig. 4C, Fig. S1B).

The intracellular labile iron pool is one of the important components of cellular iron homeostasis and reflects the cell’s capacity to regulate iron metabolism [25]. When stimulated by LPS, the intracellular Fe^2+^ iron content of OCPs increased gradually (Fig. S2), indicating increases in the labile iron pool which drives osteoclast differentiation [18]. To further investigate the effect of Lcn2 and the regulatory role of lincRNA-EPS, we add extra Lcn2 into culture environment (Fig. 4D). After LPS induction, the iron level maintained stable in WT osteoclasts regardless of *Lcn2* knockdown or Lcn2 extra supply. Without lincRNA-EPS, *Lcn2* knockdown had no effect but extra Lcn2 in environment increased the Fe^2+^ iron content. Together, the iron metabolism was regulated in short-term in WT osteoclasts after *Lcn2* knockdown or Lcn2 supply, and the active regulation mechanism helped maintaining a stable labile iron pool. On the other hand, in KO osteoclasts, intracellular iron accumulated with extra Lcn2 supply, indicating impaired efficiency in iron metabolism regulation.

Furthermore, the osteoclast differentiation was induced by LPS under conditions of *Lcn2* knockdown or extra Lcn2 supplementation (Fig. 4E). Notably, *Lcn2* knockdown resulted in an increased number of WT osteoclasts; however, these cells were smaller, with fewer nuclei and a shorter maximum diameter. Extrinsic Lcn2 reversed the effect of *Lcn2* knockdown in WT cells, promoting the formation of larger osteoclasts. In contrast, *Lcn2* knockdown or Lcn2 supply had a markedly weaker effect in KO osteoclasts. Consistent with these morphological observations, the expression of genes and proteins associated with osteoclast differentiation showed corresponding changes (Fig. S1C, D). Together with the results of gene and protein expression, this indicates that the increased secretion of Lcn2 in KO osteoclasts is not the direct cause of their enhanced osteoclast differentiation, and KO cells had a slower response to Lcn2 change.

### LincRNA-EPS overexpression enhanced iron regulatory capacity without affecting LPS-induced osteoclastogenesis

**Fig. 5:**
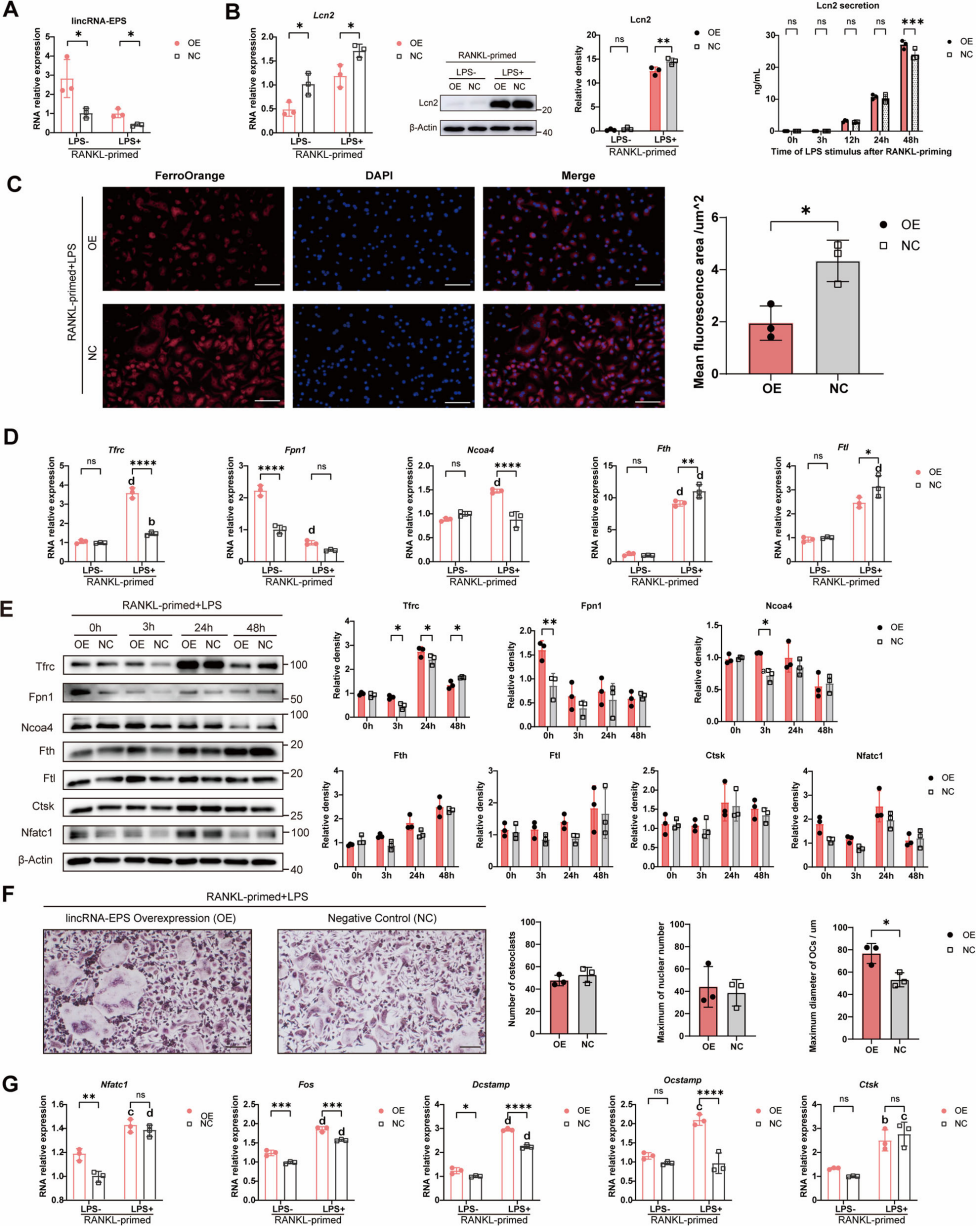
LincRNA-EPS overexpression enhanced iron regulatory capacity without affecting LPS-induced osteoclastogenesis. **A** Efficiency of lincRNA-EPS overexpression in WT OCPs (*n* = 3). **B** *Lcn2* gene expression (left panel), Lcn2 protein expression (middle panel) and secretion (right panel) post-overexpression after LPS stimulation (*n* = 3). **C** Intracellular Fe^2+^ levels detected by FerroOrange probe (*n* = 3). Scale bar: 10 μm. **D** Expression of iron metabolism-related genes post-overexpression after LPS stimulation (*n* = 3). **E** Expression of iron metabolism proteins and osteoclastogenesis key proteins after lincRNA-EPS overexpression(*n* = 3). **F** TRAP staining showing the osteoclast differentiation after lincRNA-EPS overexpression. Scale bar: 20 um. The number of osteoclasts, the maximal nuclei count and the maximum diameter were analyzed (*n* = 3). **G** The expression of osteoclastogenesis genes after lincRNA-EPS overexpression (*n* = 3). Two-way ANOVA with Tukey-Kramer test was used in **A**, **B**, **D**, **E**, **G**. Unpaired *t*-test was used in **C** and **F**. Data were presented as mean ± SD. Significance levels were denoted as follows: *P < 0.05, **P < 0.01, ***P < 0.001, ****P < 0.0001, ns refers to no significant difference. In **D**, **G**, significance levels were also denoted as a (*P* < 0.05), b (*P* < 0.01), c (*P* < 0.001) and d (*P* < 0.0001) when compared with the same group at “LPS-” timepoint.

Former studies have demonstrated that lincRNA-EPS overexpression could alleviate inflammation in tissue cells [10, 11, 13]. Building on the reported anti-inflammatory role of lincRNA-EPS and its deletion-induced hypersensitivity to LPS [9, 13], we constructed lincRNA-EPS-overexpressing (OE) OCPs via lentiviral transduction to further explore its function in cellular iron metabolism. Interestingly, despite successful overexpression, lincRNA-EPS levels were significantly reduced following LPS stimulation (Fig. 5A). Concurrently, both the gene and intracellular protein expression of Lcn2 showed a slight decrease, while the secretion of Lcn2 was not altered and even appeared elevated at 48 h post-LPS induction in lincRNA-EPS-overexpressing cells (Fig. 5B). In terms of iron metabolism, the intracellular Fe^2+^ content was decreased after lincRNA-EPS overexpression (Fig. 5C). Meanwhile, several genes related to iron transport and supply, such as *Tfrc, Fpn1, Ncoa4* were upregulated in OE osteoclasts, whereas the expression of *Fth* and *Ftl* was lower (Fig. 5D). Moreover, the expression of iron metabolism proteins was detected (Fig. 5E). Consistent with the transcriptional changes, the expression of Tfrc, Fpn1 and Ncoa4 was also altered after lincRNA-EPS overexpression. Specifically, upon LPS stimulation, the protein level of Tfrc increased during the initial 24 h and subsequently declined to normal level. This dynamic change occurred more rapidly in cells overexpressing lincRNA-EPS. Expression of Ncoa4 was modulated similarly, with a higher expression within 3 h post LPS induction in OE cells. Fpn1 was upregulated in OE OCPs before LPS induction. Together, these data indicate that the LPS-induced increase in iron metabolism was both accelerated and more transient in OE osteoclasts.

However, the expression of Ctsk and Nfatc1 showed no significant difference between OE and negative control osteoclasts, indicating comparable levels of osteoclast differentiation (Fig. 5E). Hence, we next evaluated the effect of lincRNA-EPS overexpression on osteoclast formation (Fig. 5F). TRAP staining showed that lincRNA-EPS overexpression did not impact osteoclast fusion, with similar osteoclasts numbers and maximum nuclear numbers. However, the maximum diameter of osteoclasts was increased. Meanwhile, the expression of genes involved in osteoclast fusion and enlargement—including *Fos*, *Dcstamp*, and *Ocstamp*—was upregulated in OE osteoclasts following LPS stimulation (Fig. 5G). These results suggest a complex mechanism of lincRNA-EPS in regulating osteoclast differentiation.

### Modulation of Lcn2 in vivo alleviates periodontitis bone loss in the presence of lincRNA-EPS

**Fig. 6:**
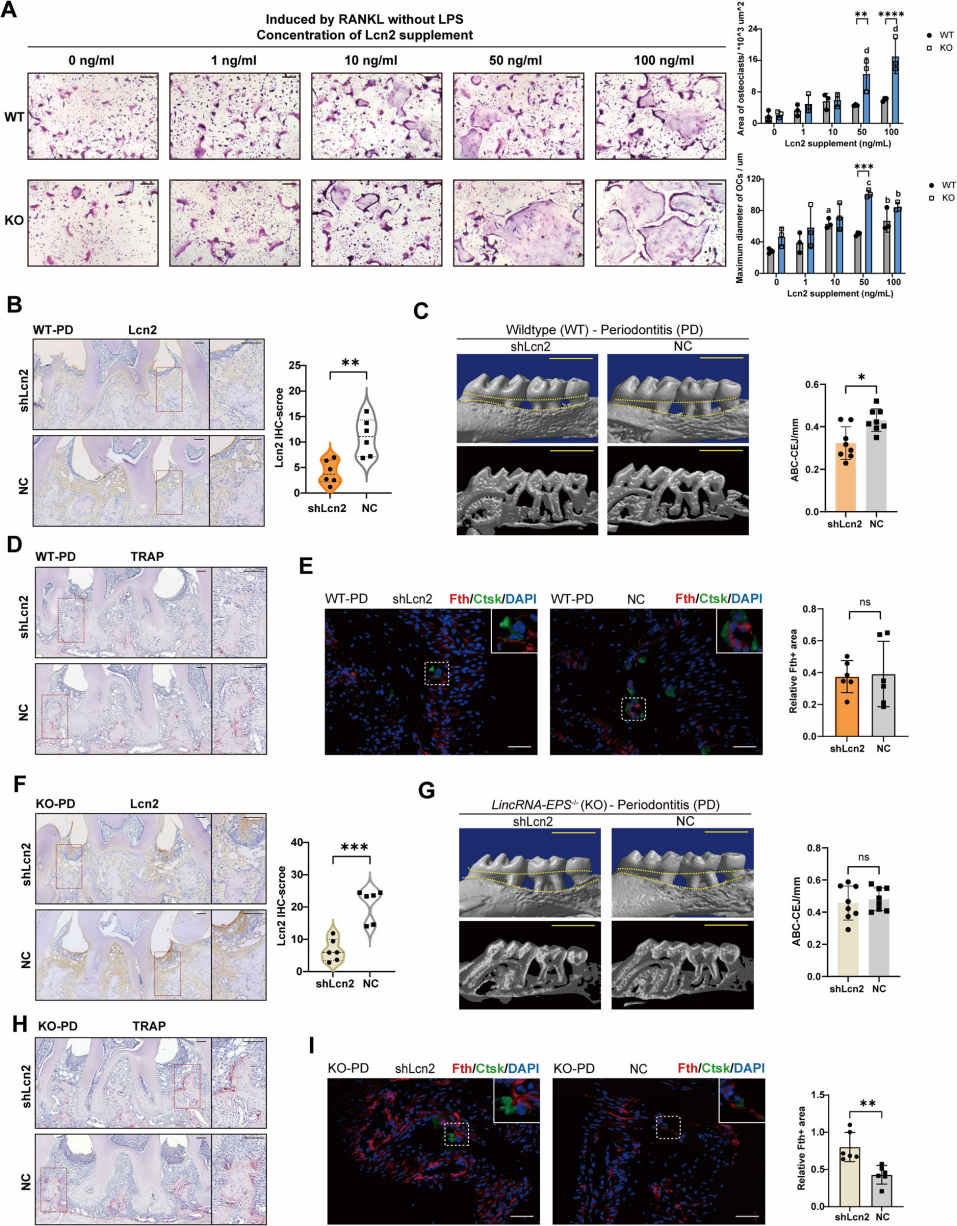
Modulation of Lcn2 in vivo alleviates periodontitis bone loss in the presence of lincRNA-EPS. **A** WT or KO osteoclasts were induced by RANKL and different concentrations of Lcn2 (*n* = 3). Scale bar: 20 um. **B** and **F** IHC staining of Lcn2 of periodontal tissues (*n* = 6). Scale bar: 100 μm. **C** and **G** Micro-CT images of alveolar bone resorption in *Lcn2*-knockdown WT mice and KO mice (*n* = 8). Scale bar: 1 mm. **D** and **H** TRAP staining of periodontal tissues. Scale bar: 100 μm. **E** and **I** IF staining of ferritin heavy chain and Ctsk. The inset in the upper-left corner shows a 2× magnified view (*n* = 6). Scale bar: 50 μm. Data were presented as mean ± SD. Two-way ANOVA with Tukey–Kramer test was used in **A**. Unpaired *t*-test was used in **B**, **E**, **F**, and **I**. Paired *t*-test was used in **C** and **G**. Significance levels were denoted as follows: *P < 0.05, **P < 0.01, ***P < 0.001, ****P < 0.0001, ns refers to no significant difference. In **A**, significance levels were also denoted as a (*P* < 0.05), b (*P* < 0.01), c (*P* < 0.001) and d (*P* < 0.0001) when compared with the data in “0 ng/mL” group in the same cell type.

As shown in Fig. 3A, Lcn2 is predominantly expressed in the gingival epithelium and connective tissues in periodontitis lesions. Therefore, under in vivo conditions, it is more likely that tissue-derived Lcn2 in the microenvironment, rather than that produced by osteoclasts themselves, influences osteoclast differentiation. To examine this hypothesis, different concentrations of Lcn2 were added to RANKL-induced cultures of WT and KO BMDMs in the absence of LPS (Fig. 6A). The results showed that high concentrations of Lcn2 promoted osteoclast differentiation, and this promoting effect was more pronounced in KO cells.

Finally, the influence of lincRNA-EPS or Lcn2 in vivo was investigated on LPS-induced periodontitis mice model using lincRNA-EPS overexpression or *Lcn2* knockdown lentivirus (Fig. S3). As shown in Micro-CT, local lincRNA-EPS overexpression alleviated bone resorption (Fig. S4A, B). However, Lcn2 expression and Fth expression in periodontitis tissue was not changed obviously (Fig. S4C, D), indicating that lincRNA-EPS may also regulate tissue inflammation through other mechanisms.

On the other hand, similarly with the results of in vitro osteoclast differentiation, *Lcn2* knockdown caused different effects on WT and *lincRNA-EPS*^*-/-*^ (KO) mice. The expression of Lcn2 in periodontal tissue was decreased successfully in both WT and KO mice (Fig. 6B, F). However, on WT mice, *Lcn2* knockdown decreased bone resorption and osteoclast activity (Fig. 6C, D), while no such effect was seen on KO mice (Fig. 6G, H). Furthermore, there was no significant change in Fth expression on WT mice but more Fth expression on KO mice following *Lcn2* knockdown (Fig. 6E, I). These results suggested that modulating the expression of Lcn2 in the microenvironment could attenuate osteoclast differentiation and function in the presence of lincRNA-EPS.

## Discussion

Periodontitis is a typical inflammatory bone resorption disease. The mechanism of osteoclast differentiation under inflammatory environment is complex and always shows difference with the process under physiological conditions [26–28]. Here, our research focused on the regulation of lincRNA-EPS on osteoclast differentiation under LPS induction, and revealed its effect on iron metabolism. LincRNA-EPS acted as a “brake” on excessive iron metabolism and iron ion accumulation, thus helped stabilized intracellular iron homeostasis and decreased osteoclast formation under inflammatory environment.

The relationship between osteoclast formation and iron metabolism plays a crucial role in studies of bone diseases such as osteoporosis. This research found active iron metabolism and iron ion accumulation in LPS-induced osteoclast. In accordance with this result, previous research indicates that iron metabolism regulation significantly influences the generation and function of osteoclasts. For example, hepcidin promotes the proliferation and differentiation of osteoclast precursor cells by downregulating ferroportin expression, thereby increasing intracellular iron levels [29]. Iron chelators, which reduce iron overload in cells, could inhibit osteoclast differentiation and promote osteoblast differentiation, exhibiting bone-protective effects across multiple osteoporosis models [30, 31].

Our study found that the deficiency of lincRNA-EPS promoted LPS-induced osteoclastogenesis and iron metabolism, and Lcn2 was remarkably upregulated in this process. This finding guided our attention on Lcn2. As a secretion protein binding with iron ions, its biological function depends on its location and form [32]. Several researches have studied the role of Lcn2 in body functions, including iron homeostasis. Lcn2 reduces intracellular iron level in some studies. In pancreatic ductal adenocarcinoma cells, Lcn2—regulated by the transcription factor Nuclear protein-1 —reduces intracellular iron concentration, thereby inhibiting ferroptosis [33]. Deficiency in Lcn2 leads to iron homeostatic imbalance and exacerbates endotoxin-induced sepsis, demonstrating its antioxidant effects in regulating free iron levels in the body [34]. In glioblastoma cells, Lcn2 elevation upon NF-κB pathway activation exerts iron-chelating effects that mitigate ferroptosis [35]. Some studies have also found that Lcn2 increases cellular iron levels. In osteoblasts, increased Lcn2 induces intracellular iron overload, triggering cellular apoptosis [36]. In murine lung cancer models, Lcn2 is primarily produced by neutrophils and released into tissues, where it promotes ferroptosis in adipose and skeletal tissues [37]. Altogether, the effect of Lcn2 on cellular iron homeostasis is complicated and might be finely regulated.

In this study, Lcn2 knockdown and exogenous Lcn2 supplementation experiments suggested that Lcn2 could promote osteoclast fusion and enlargement under inflammatory environment. This is a new perspective for the role of Lcn2 in osteoclastogenesis. Previously, a few studies focused on the effect of Lcn2 on osteoclast differentiation, but the results are controversial. Mattia Capulli et al. reported that Lcn2-knockout mice exhibited reduced bone mass, decreased osteoblast number and activity, but no significant changes in osteoclast phenotype [38]. In contrast, Delfina Costa et al. found that Lcn2 overexpression enhanced osteoclast differentiation [39]. Conversely, Hyun-Ju Kim and Jiah Yeom et al. demonstrated that both endogenous and exogenous Lcn2 suppressed osteoclastogenesis [40, 41]. However, these studies investigate the effect of Lcn2 in healthy mice or environment. Different from previous studies, we focused on LPS-induced osteoclast differentiation, which was more similar with the osteoclastogenesis in periodontitis environment. Unlikely in physiological condition, under the stimulation with LPS, Lcn2 expression and secretion increased, participating in regulating iron metabolism and promoting osteoclastogenesis.

One unexpected finding was the inconsistency between intracellular iron ion level and osteoclast differentiation. In OCPs treated with lincRNA-EPS overexpression lentivirus, the Fe^2+^ content, which consists the intracellular labile iron pool, significantly decreased, while osteoclastogenesis was not influenced. In WT OCPs under Lcn2 knockdown or extrinsic Lcn2 supplementation, the Fe^2+^ levels were kept nearly constant, while the size of osteoclasts changed. As for *lincRNA-EPS*^*-/-*^ OCPs under Lcn2 knockdown and Lcn2 supplementation, the situation was reversed. This rather intriguing result might be explained by the dynamic regulation of intracellular iron levels [25]. The regulation of cellular and systemic iron homeostasis is achieved through a series of complex signaling pathways. These include the iron regulatory protein-iron response element network, the ferritin autophagy pathway mediated by Ncoa4, the prolyl hydroxylase domain -hypoxia-inducible factor axis, and the NRF2 regulatory center, etc [42, 43]. These mechanisms work together to ensure iron uptake, transport, and release within cells, as well as mobilization of stored iron reserves, thereby meeting cellular iron demands while limiting the potential adverse effects of iron-mediated oxidative damage and ferroptosis. The osteoclast differentiation requires higher iron ion supply than physiological conditions [44, 45]. Iron ions directly participate in the metabolic activities of osteoclasts, and also generate reactive oxygen species through *Fenton* reaction, which next activate signaling pathways of osteoclastogenesis [46]. That is, the iron ion level in osteoclasts is associated with the its uptake, transportation, consumption and ferritinophagy. The high Fe^2+^ level may indicate both high iron supply or low iron consumption. Therefore, rather than the levels of iron ions, the stability of intracellular labile iron pool deserves more attention, as it reflects normal cellular capacity for iron metabolism regulation. This stability enables cells to dynamically modulate iron metabolism in response to external stimuli, thereby meeting variable iron demands and preventing iron overload and subsequent cellular damage [47, 48].

Based on our findings, LPS induced Lcn2 expression and secretion as well as enhanced iron metabolism and osteoclastogenesis. Lcn2 plays a dual role as both an indicator and a regulator of iron metabolism. Surprisingly, the iron metabolic changes caused by Lcn2 knockdown occurred more slowly in *lincRNA-EPS*^*-/-*^ cells than in WT cells, evidenced by later protein/gene expression changes and unstable Fe^2+^ levels. In contrast, lincRNA-EPS overexpression enhanced iron metabolism regulation capacity, leading to an accelerated response to external stimuli. This indicates that lincRNA-EPS helps cells regulate their iron metabolism levels in response to environmental changes and promptly return to iron homeostasis. However, the inductive effect of RANKL or LPS is more pronounced. Under inflammatory conditions, where lincRNA-EPS expression is significantly downregulated, its overexpression fails to alter Lcn2 secretion or overall osteoclast differentiation. Taken together, these findings imply that lincRNA-EPS enables adaptive regulation of cellular iron metabolism to environmental fluctuations, ensuring that iron homeostasis is maintained to meet physiological needs while averting pathological overactivation or overload.

This may explain why *Lcn2* knockdown and Lcn2 supplementation had minimal effect on osteoclast differentiation in *lincRNA-EPS*^*-/-*^ cells. The critical period for osteoclast differentiation occurs within 24 h post-LPS induction, coinciding with the peak of NFATc1 protein (Figs. S1D and 5D). Due to their slower iron metabolism regulation, KO cells remained under the dominant influence of the initial RANKL priming and subsequent LPS stimulation during this decisive period. Consequently, the interventions failed to alter the osteoclast differentiation outcome.

The role of Lcn2 in promoting osteoclast function and the regulatory effect of lincRNA-EPS were validated in the in vivo models. On one hand, *Lcn2* knockdown alleviated bone resorption in WT mice without affecting Fth in surrounding cells, but this effect was reversed in *lincRNA‑EPS*^*‑/*‑^ mice. On the other hand, lincRNA‑EPS overexpression reduced osteoclast activity and bone resorption without altering Lcn2 or Fth expression. This again demonstrates the dominant role of tissue inflammation in osteoclastogenesis [49]. Prior study showed that lincRNA‑EPS^‑/‑^ mice exhibit heightened periodontal inflammation, while its overexpression reduces inflammation, which is consistent with our findings [11]. Thus, Lcn2 knockdown may fail to counteract osteoclast promotion driven by inflammatory signals in *lincRNA‑EPS*^*‑/*‑^ mice. The difference in Fth expression between WT and KO mice upon Lcn2 knockdown also warrants discussion. In WT mice, unchanged Fth suggests stable tissue iron storage, likely due to metabolic stabilization by the 2‑week endpoint under lincRNA‑EPS regulation. In *lincRNA‑EPS*^*‑/*‑^ mice, impaired iron metabolism and lower Lcn2 lead to iron imbalance; inflammation‑induced iron accumulation is stored as Fth, explaining its elevated expression.

There are some limitations in this study. First of all, the relationship between lincRNA-EPS and iron metabolism was not elucidated. Whether lincRNA-EPS directly regulates the production of Lcn2 or other iron metabolism proteins and how it modulates the cellular response to extracellular Lcn2 remain unclear. Future investigation is needed to explore the relationship between them. Secondly, rather than number of osteoclasts, the size and nuclei of osteoclasts changed obviously under lincRNA-EPS overexpression or Lcn2 alterations, indicating the fusion and enlargement of osteoclast were influenced by these treatments and iron metabolism. Nevertheless, the specific stage(s) of osteoclast differentiation affected by iron metabolism remain undefined in this study. Furthermore, the methodologies employed for detecting iron metabolism in this study were relatively rudimentary. The assessment of iron metabolism relied on endpoint measurements of total iron content, which lacks spatiotemporal resolution to dissect compartment-specific iron dynamics during osteoclastogenesis. Also, Lcn2 exists in distinct variants that may confer divergent cellular functions. Future studies require the application of more precise iron assessment techniques combined with stage-specific dissection of osteoclast differentiation to elucidate the mechanisms by which Lcn2 modulates inflammatory osteoclast differentiation through iron metabolism.

In conclusion, we identified the critical role of lincRNA-EPS in regulating osteoclastogenesis under inflammatory environment, and further dissects its mechanisms in bone resorption. Under inflammatory conditions, Lcn2 expression triggers disruption of iron homeostasis in osteoclasts, and lincRNA-EPS facilitates the adaptive regulation of iron metabolism to meet cell demands, preventing its excessive and prolonged activation, and thereby mitigating the excessive differentiation of osteoclasts. This study not only proposes that Lcn2 modulates the functionality of osteoclasts by reprogramming iron metabolism, but also provides a theoretical foundation for lncRNA-mediated biological regulation of osteoclasts.

## Materials and methods

### LPS-induced periodontitis mouse model

The sample size for the in vivo experiments was determined by a power analysis, with a statistical power set at 80% and a significance level of 5% [50]. Based on preliminary experiments indicating a standard deviation of 10–15%, and with the aim of detecting a minimum intergroup difference of 30%, a minimum of 6 mice per experimental group was calculated. In this study, 6–8 mice per group were ultimately used, which proved sufficient to yield a statistically significant difference. Animals were allocated to different groups using a random number method, and the researchers performed the experiments and data analysis in a blinded manner.

*LincRNA-EPS*^*-/-*^ mice were generated by Shanghai Model Organisms Center, lnc (Shanghai, China).

Periodontitis was experimentally induced in 8-week-old male *lincRNA-EPS*^*-/-*^ (KO) and wildtype (WT) C57BL/6 mice via silk thread ligation saturated with lipopolysaccharide (LPS, Sigma, L2880). In each mouse, a periodontitis model was established on one side of the maxilla, while the contralateral side served as a healthy control. After 14 days, mice were sacrificed and the alveolar bone resorption was quantified using micro-computed tomography.

Ethical approval was obtained from the Ethics Review Board of the Affiliated Stomatology Hospital of Tongji University (NO. [2022]-DW-29) and were performed in compliance with ARRIVE guidelines.

### Tissue sections and staining

The periodontal tissues were collected and fixed with 4% paraformaldehyde (Beyotime, P0099) for 2 days, decalcified with 10% ethylenediaminetetraacetic acid solution (Sangon Biotech, A500895) and then embedded in paraffin. The paraffin sections were deparaffinized and hydrated for further staining. Tartrate resistant acid phos-phatase (TRAP) staining was completed under the kit instructions (Servicebio, G1050). For immunohistochemical (IHC) and immunofluorescence (IF) staining, the sections were subjected to antigen repair (Beyotime, P0084), endogenous peroxidase inactivation (MXB biotechnologies, SP KIT-A2), blocking (Beyotime, P0260), and then incubated with Lcn2 (Proteintech, 30576-1-AP) or Fth (Abclonal, A19544) primary antibodies at 4 °C overnight. After washing with PBS, sections were incubated with corresponding secondary antibodies at 37 °C for 1 h. For IHC, sections were incubated with streptomyces anti-biotin protein-peroxidase (MXB biotechnologies, SP KIT-D2) and DAB solution (KeyGEN biotech, KGP1045-20), followed by nuclear counterstaining by hematoxylin. For IF, nuclei were stained by DAPI (Beyotime, C1006). Images were captured with a high-resolution digital microscope (Nikon). DAB staining intensity was measured using Image J, and the IHC score was calculated based on both staining intensity and stained area. Fluorescence intensity was quantified through Image J software.

### Osteoclast differentiation in vitro

For in vitro osteoclastogenesis assays, bone marrow-derived macrophages (BMDMs) isolated from both genotypes and cultured in α-MEM medium (Hyclone, SH30265.01) containing 10% FBS (Gibco, A5669701) and 1% penicillin/streptomycin (Hyclone, SV30010) overnight. Next, the nonadherent cells were collected, counted and plated according to experiment needs in complete medium supplemented with macro-phage colony-stimulating factor (M-CSF) (Sino Biological, 51112-MNAH), receptor activator for nuclear factor-κB ligand (RANKL) (R&D Systems, 462-TEC). LPS (Sigma, L2880) was applied after RANKL-priming for two days when mimicking the inflammatory environment. Osteoclast formation was assessed through tartrate TRAP staining (Sigma, 387 A). For the experiment assessing bone resorption capability, BMDMs were cultured on bone slices (600 μm thick, Amizona, AMB1003, China) with M‑CSF, RANKL, and LPS as inducers in the culture medium. After 12 days of induction, cells on the bone slices were removed by sonication. The morphology of bone resorption pits was observed by electron microscopy, with their area quantified. Throughout the culture, no form of contamination was observed or detected.

### RNA extraction and qRT-PCR

Total RNA was extracted with RNAiso Plus reagent (Takara, 9109) according to instructions. Reverse transcription was performed using cDNA Synthesis SuperMix (Yeasen, 11141ES10), and then qRT-PCR was performed using SYBR Green Reagent (Yeasen, 11201ES08) with corresponding primers (Table S1). The expression levels of genes were normalized to β-actin levels.

### Protein extraction and western blotting

Total proteins were extracted using RIPA Lysis Buffer (Beyotime, P0013B) supplemented with protease and phosphatase inhibitor cocktail (Beyotime, P1045). Proteins were boiled for 10 minutes with sample loading buffer (Beyotime, P0015) and stored at −80 °C. For Western Blotting, proteins were separated through SDS-PAGE and transferred to NC membranes according to instrument’s introductions (Epizyme Biotech, PG112, PG113 and WJ003). The membranes were then blocked with 5% non-fat milk and incubated with primary antibodies at 4 °C overnight. The next day, the membranes were washed and incubated with secondary antibodies at room temperature for 1 h. Afterwards, ECL Western Blotting Substrate were used to capture protein bands (Affinity, KF8005). The details of antibodies were summarized in Table. S2. Protein expression levels were quantified by grayscale analysis using ImageJ software. Full and uncropped western blots are provided in supplemental materials.

### Enzyme-linked immunosorbent assay (ELISA)

Cell culture supernatant was collected. The secretion level of Lcn2 was detected by ELISA following the protocols of the kit (Elabscience, E-EL-M0828).

### RNA and protein sequencing analysis

After required treatments, total RNA and protein of pre-osteoclasts or mature osteoclasts were extracted as described previously. RNA and protein quality assessment and sequencing were performed at Majorbio (Shanghai Majorbio Bio-pharm Technology Co.,Ltd). Data analysis was performed using the Majorbio Cloud (www.majorbio.com).

### Gene overexpression and knockdown

LincRNA-EPS overexpression and *Lcn2* knockdown were achieved through lentivirus. Following lentivirus were purchased from OBiO Technology (Shanghai) Corp., Ltd: lincRNA-EPS overexpression lentivirus (pASLenti-pA-Ttc39aos1-CMV-EF1-EGFP-WPRE) and negative control (pASLenti-pA-MCS-CMV-EF1-EGFP-WPRE), *Lcn2* knockdown lentivirus (pCLenti-U6-shRNA(Lcn2)-CMV-EGFP-WPRE) and negative control (pCLenti-U6-shRNA(NC)-CMV-EGFP-WPRE). For lentivirus transfection, BMDMs were seeded in 96-well or 6-well plates overnight and then infected with corresponding lentivirus at a multiplicity of 30. After 12 ~ 16 h, transfected BMDMs were induced by RANKL for further tests. For gene overexpression and knockdown in vivo, the lentivirus was injected to the top of alveolar crest 3 days before silk-ligation. Additionally, the overexpression or knockdown lentivirus was injected into one side of the maxilla, with the other side receiving the negative control lentivirus.

### Iron content assessment

OCPs were induced with LPS, and the intracellular Ferrous ion content was detected by FerroOrange (Dojindo, F374) and observed by fluorescence microscope. Fluorescence intensity was quantified through Image J software.

### Statistics

All in vitro experiments were performed with a minimum of three biological replicates. For in vivo experiments, each group consisted of 6 to 8 mice. Exact sample sizes for each group/condition are indicated in the corresponding figure legends. Each data point represents one biological replicate from an individual mouse or independent culture.

Data were shown as mean ± standard deviation (SD). Differences analysis and figure drawing were performed by the software Prism 9 (Chicago, USA). Prior to statistical analysis, the normality of the distribution and homogeneity of variance were assessed. Statistical analyses were conducted as follows: two-sided paired or unpaired *t*-tests were used for comparisons between two groups; one-way analysis of variance (ANOVA) with Tukey–Kramer post hoc testing was applied for comparisons among multiple groups; and two-way ANOVA with appropriate multiple comparisons was used for multi-factor designs involving multiple groups. Differences were considered statistically significant when *P* < 0.05. Significance levels were denoted as follows: *P < 0.05, **P < 0.01, ***P < 0.001, ****P < 0.0001, “ns” indicates no significant difference.

## Supplementary information

Supplement materials

Original Western Blots

## Data Availability

The mRNA sequencing datasets generated during the current study have been deposited in the NCBI Gene Expression Omnibus (GEO) database (GSE305332). The mass spectrometry proteomics data have been deposited to the ProteomeXchange Consortium (https://proteomecentral.proteomexchange.org) via the iProX partner repository with the dataset identifier PXD071554. Other data in this study are available from the corresponding author on reasonable request.
