# Supplementary material for: LincRNA-EPS alleviates osteoclastogenesis under inflammatory microenvironment through preventing excessive iron metabolism
Source: Cell Death Dis. 2026 Apr 3;17(1):444. doi: 10.1038/s41419-026-08716-y (PMC13172043; doi:10.1038/s41419-026-08716-y)
Supplement: Supplementary file 1 — Supplement materials [file 41419_2026_8716_MOESM1_ESM.docx]

**Supplementary materials**

**LincRNA-EPS** **alleviates osteoclastogenesis under inflammatory microenvironment through preventing excessive iron metabolism**

Jin Wang^1*^, Yabing Wang^2*^, Zhanwei Zhang^1^, Xin Wang^1^, Jiansheng Su^1#^

1 Shanghai Engineering Research Center of Tooth Restoration and Regeneration & Tongji Research Institute of Stomatology & Department of Prosthodontics, Shanghai Tongji Stomatological Hospital and Dental School, Tongji University, Shanghai, 200072, China

2 Shanghai Engineering Research Center of Tooth Restoration and Regeneration & Tongji Research Institute of Stomatology & Department of Endodontics, Shanghai Tongji Stomatological Hospital and Dental School, Tongji University, Shanghai, 200072, China

* These authors contributed equally to this work.

# Corresponding author: Jiansheng Su

Address: Shanghai Tongji Stomatological Hospital, No. 399, Yanchang Road (M), Jing'an District, Shanghai, 200072, China.

Telephone: +86-13611669191

Email: sjs@tongji.edu.cn

**This file includes:**

**Figure. S1 to S4.**

**Table S1 to S2.**


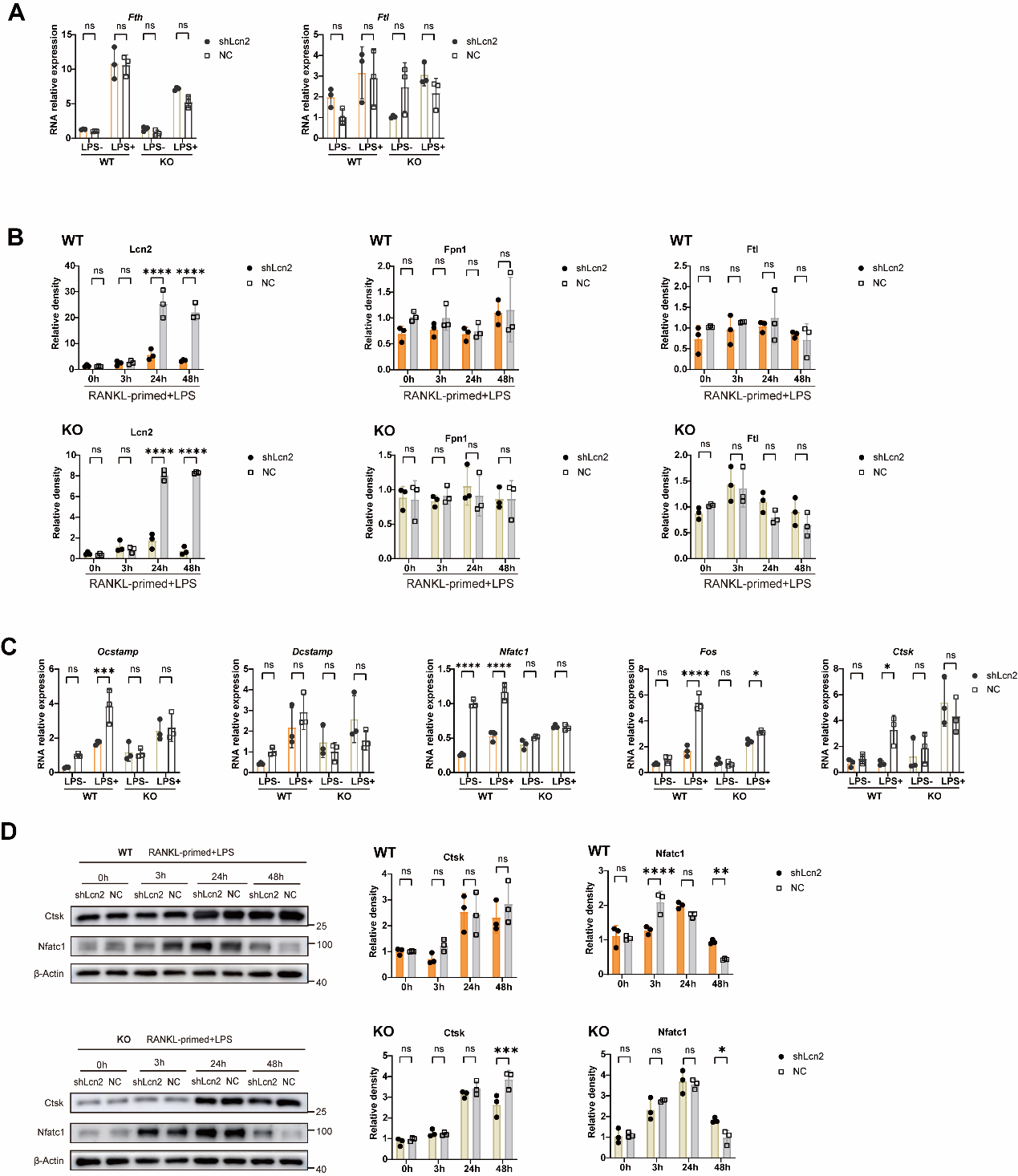


**Fig. S1 The supplementary data of genes and proteins expression after *Lcn2* knockdown in WT and KO cells.** **A** *Fth* and *Ftl* genes expression after *Lcn2* knockdown (n=3). **B** The quantification of Lcn2, Fpn1 and Ftl protein expression under RANKL-primed and LPS stimulation after Lcn2 knockdown. **C** and **D** The expression of osteoclastogenesis genes (**C**) and proteins (**D**) under RANKL-primed and LPS stimulation after Lcn2 knockdown (n=3). Data were presented as mean ± SD. Significance levels were denoted as follows: **P* < 0.05, **​​*P* < 0.01, ***​​*P* < 0.001, ****​​​​*P* < 0.0001, ns refers to no significant difference.


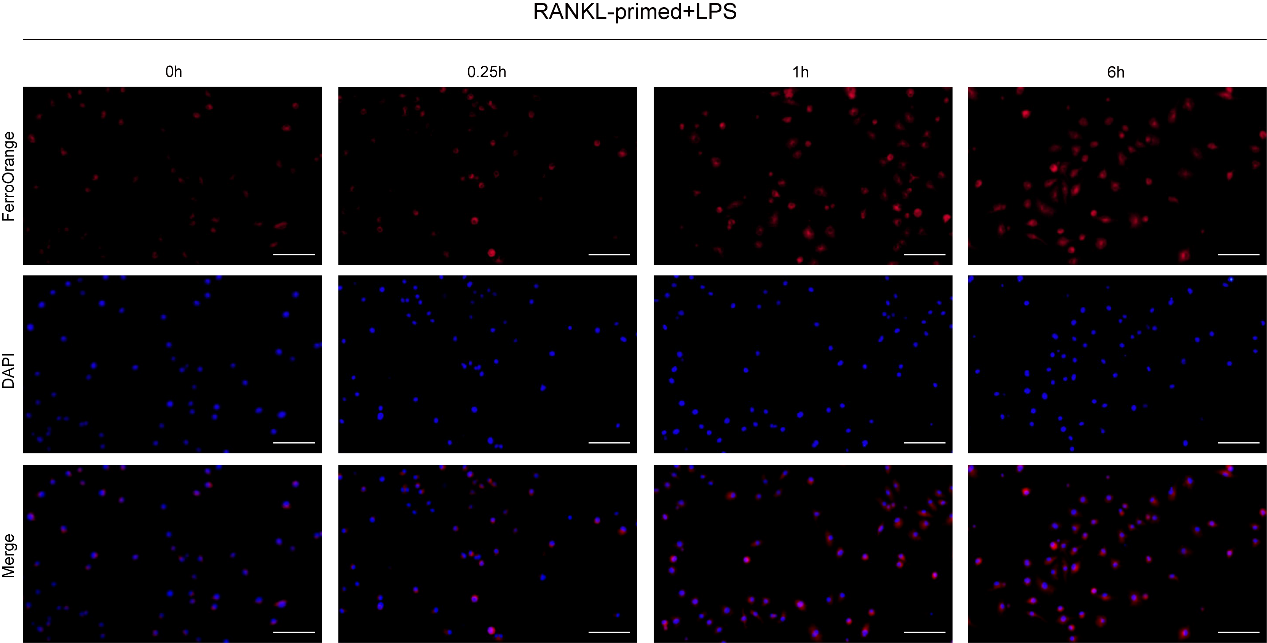


**Fig. S2** Intracellular Fe^2+^ levels detected by FerroOrange probe of osteoclast precursors after different time of LPS stimulation. Scale bar: 10 μm.


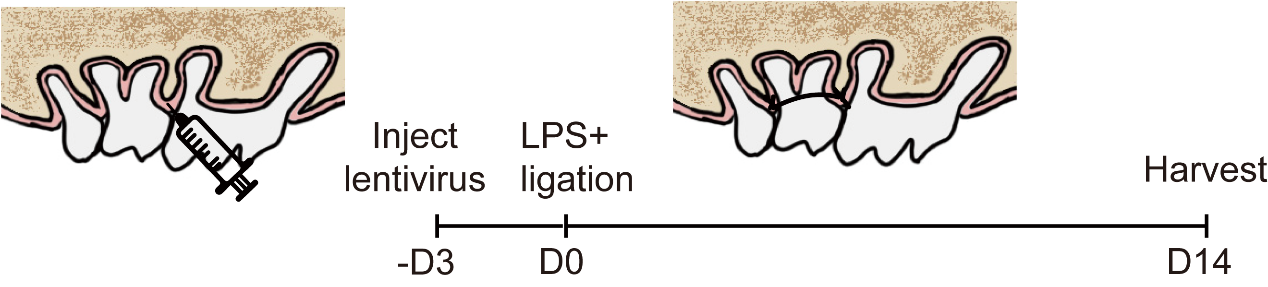


**Fig. S3** In vivo application of lincRNA-EPS overexpression and *Lcn2* knockdown lentivirus on LPS-induced periodontitis mice model.


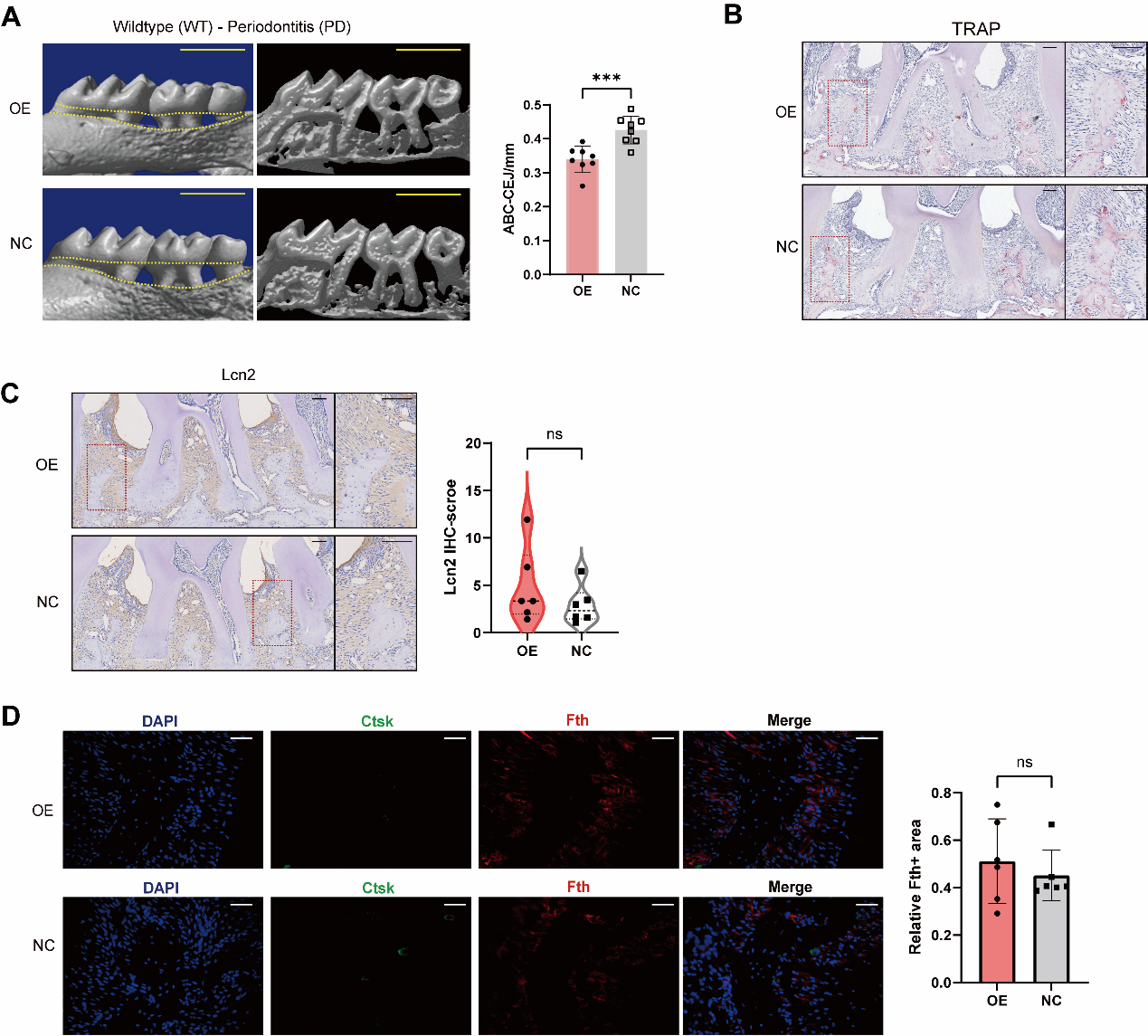


**Fig. S4** **Modulation of lincRNA-EPS *in vivo* and its effect. A** Micro-CT images of alveolar bone resorption in​​ lincRNA-EPS overexpression WT mice (n=8). Scale bar: 1 mm. **B ​**TRAP staining of periodontal tissues. Scale bar: 100 μm. **C** IHC staining of Lcn2 of periodontal tissues (n=6). Scale bar: 100 μm.​​ **D** IF staining of ferritin heavy chain and Ctsk (n=6). Scale bar: 50 μm. Data were presented as mean ± SD. Unpaired *t*-test was used in **C** and **D**. Paired *t*-test was used in **A**. Significance levels were denoted as follows: **P* < 0.05, **​​*P* < 0.01, ***​​*P* < 0.001, ****​​​​*P* < 0.0001, ns refers to no significant difference.

**Table S1. Primers sequences used for qRT-PCR**

| **Gene name** | **Forward sequence** | **Reverse sequence** |
| --- | --- | --- |
| *β-Actin* | GGCTGTATTCCCCTCCATCG | CCAGTTGGTAACAATGCCATGT |
| *LincRNA-EPS* | ATGTGTATTAGAGTTTTGCCT | TCTTTTCAAGCCCATATGTGA |
| *Ctsk* | TCCAGTTTTACAGCAGAGGTGT | CAGTGCTTGCTTCCCTTCTG |
| *Mmp9* | CTGGACAGCCAGACACTAAAG | CTCGCGGCAAGTCTTCAGAG |
| *Dcstamp* | TACGTGGAGAGAAGCAAGGAA | ACACTGAGACGTGGTTTAGGAAT |
| *Ocstamp* | CTGTAACGAACTACTGACCCAGC | CCCAGGCTTAGGAAGACGAAG |
| *Fos* | CGGGTTTCAACGCCGACTA | TTGGCACTAGAGACGGACAGA |
| *Lcn2* | CCACCACGGACTACAACCAG | AGCTCCTTGGTTCTTCCATACA |
| *Tfrc* | ATGCCGACAATAACATGAAGGC | ACACGCTTACAATAGCCCAGG |
| *Fth* | CAAGTGCGCCAGAACTACCA | GGAAGATTCGGCCACCTCG |
| *Ftl* | ATGGGCAACCATCTGACCAA | GCTGCCTAGTGGCTTGAGAGG |
| *Fpn1* | ACAAACAAGGGGAGAACGC | ATGACGGACACATTCTGAACCA |
| *Ncoa4* | GCTCAGCAGCTCTATTGGC | CAAACTGCCCAGTCTCTCCA |
| *Slc7a11* | GGCACCGTCATCGGATCAG | CTCCACAGGCAGACCAGAAAA |
| *Hmox1* | GCCGAGAATGCTGAGTTCATG | TGGTACAAGGAAGCCATCACC |
| *Nrf2* | AGTGGATCTGCCAACTACTC | CATCTACAAACGGGAATGTCTG |
| *Acsl4* | CCACACTTATGGCCGCTGTT | GGGCGTCATAGCCTTTCTTG |
| *Gpx4* | GCCTGGATAAGTACAGGGGTT | CATGCAGATCGACTAGCTGAG |

**Table S2. Antibodies used for WB, IHC and IF**

| **Antibodies** | **Manufacturer** | **Catalog Number** |
| --- | --- | --- |
| Nfatc1 | Affinity Biosciences | DF6446 |
| Ctsk | Santa Cruz Biotechnology | sc-48353 |
| Lcn2 | Proteintech Group | 30576-1-AP |
| Tfrc | ABclonal Technology | A25900 |
| Fpn1 | ABclonal Technology | A14884 |
| Ncoa4 | ABclonal Technology | A5695 |
| Fth | ABclonal Technology | A19544 |
| Ftl | Abmart | T56955 |
| β-Actin | Affinity Biosciences | T0022 |
