# Supplementary material for: LincRNA-EPS alleviates osteoclastogenesis under inflammatory microenvironment through preventing excessive iron metabolism
Source: Cell Death Dis. 2026 Apr 3;17(1):444. doi: 10.1038/s41419-026-08716-y (PMC13172043; doi:10.1038/s41419-026-08716-y)
Supplement: Supplementary file 2 — Original Western Blots [file 41419_2026_8716_MOESM2_ESM.docx]

| Image in figure | Original image | Original full image  (with same handwritten number) | β-Actin of original full image |
| --- | --- | --- | --- |
| Fig. 1J  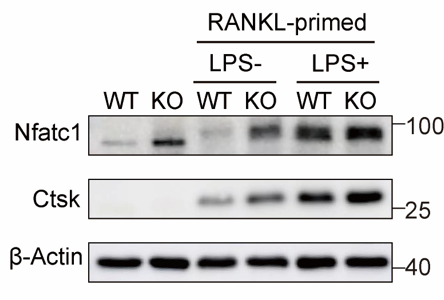 | Nfatc1  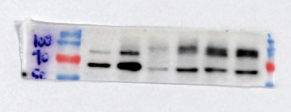 | 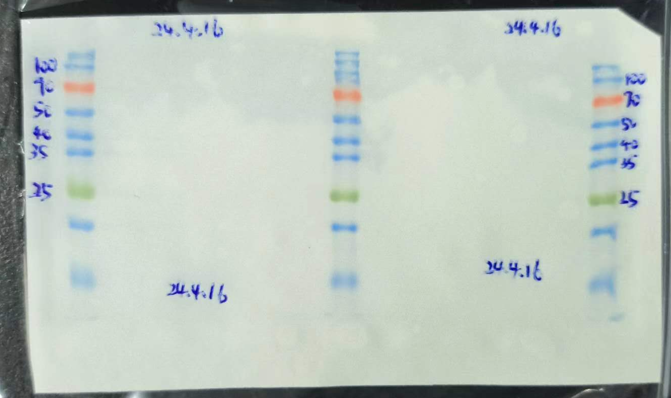 | 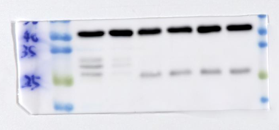 |
|  | Ctsk  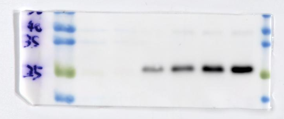 |  |  |
|  | β-Actin  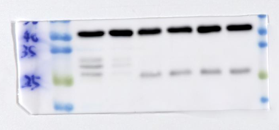 |  |  |

| Image in figure | Original image | Original full image  (with same handwritten mark) | β-Actin of original full image |
| --- | --- | --- | --- |
| Fig. 3G  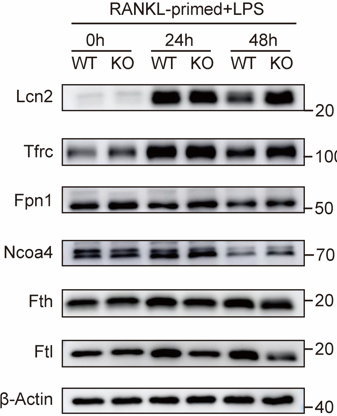 | Lcn2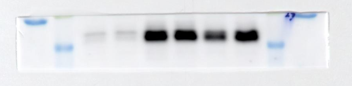 | 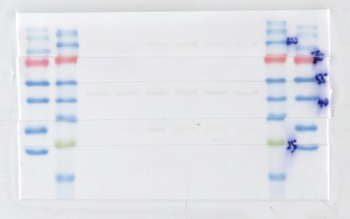 | 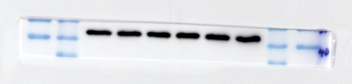 |
|  | β-Actin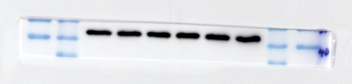 |  |  |
|  | Fpn1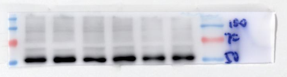 | 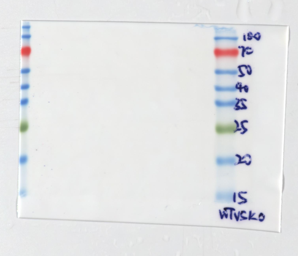 | 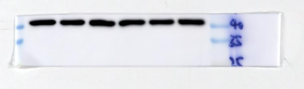 |
|  | Ncoa4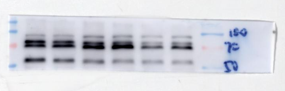 |  |  |
|  | Fth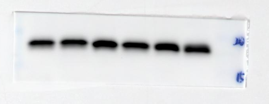 | 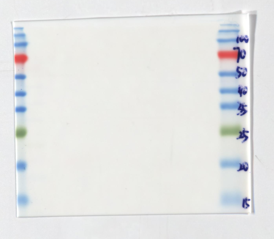 | 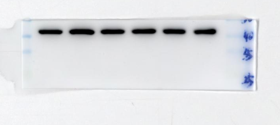 |
|  | Ftl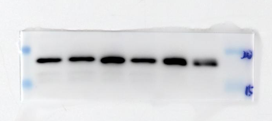 |  |  |
|  | Tfrc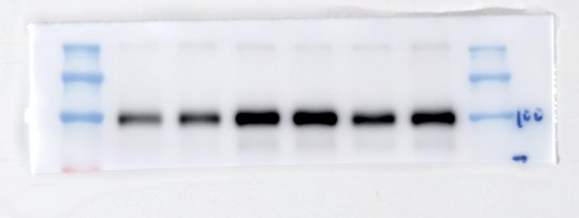 | 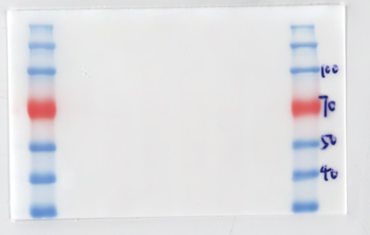 | 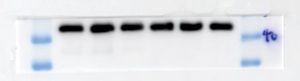 |

| Image in figure | Original image | Original full image  (with same handwritten number) | β-Actin of original full image |
| --- | --- | --- | --- |
| Fig.4B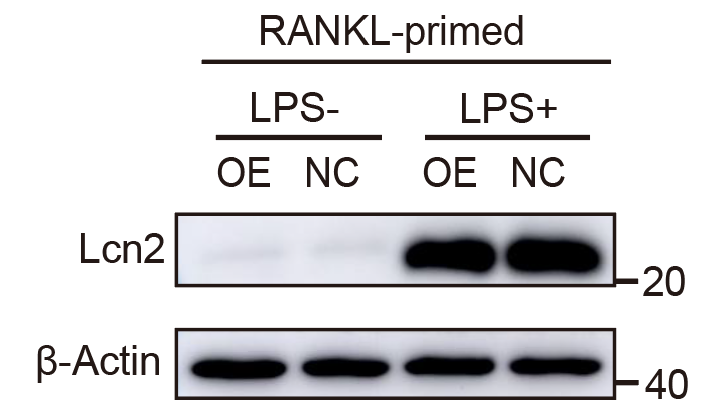 | Lcn2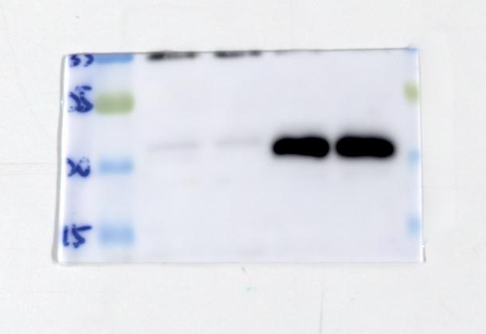 | 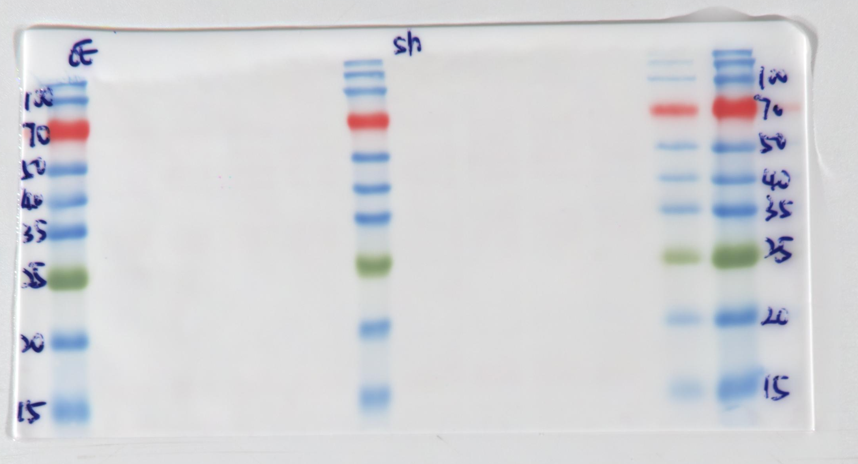 | 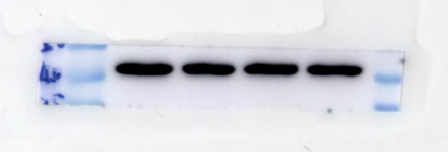 |
|  | β-Actin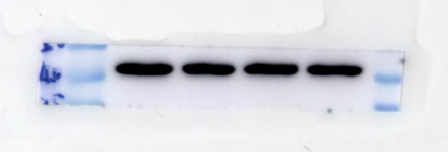 |  |  |

| Image in figure | Original image | Original full image  (with same handwritten number) | β-Actin of original full image |
| --- | --- | --- | --- |
| Fig.4E  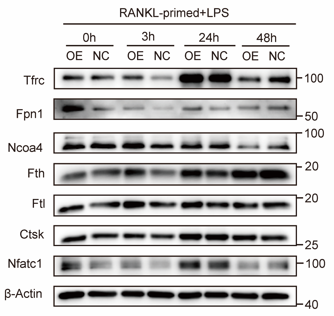  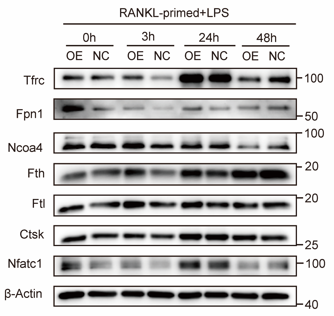 | **Tfrc**  color+chemi: 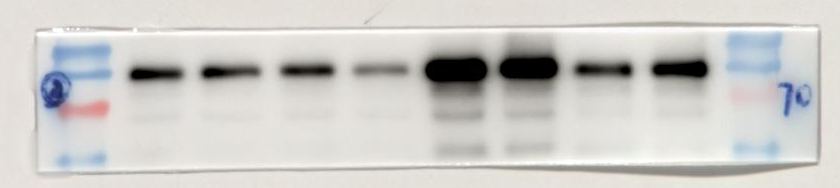chemi: 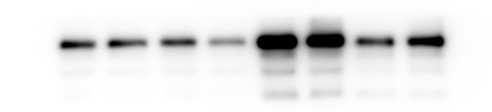 | 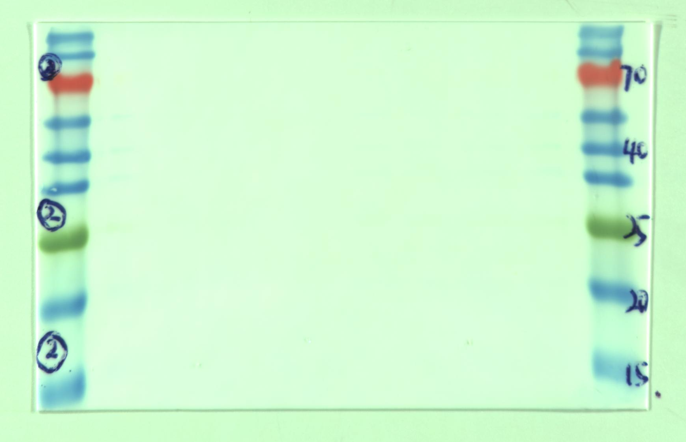 | 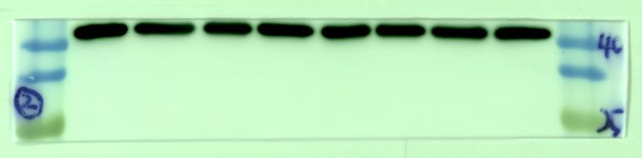 |
|  | **Fpn1**  color+chemi: 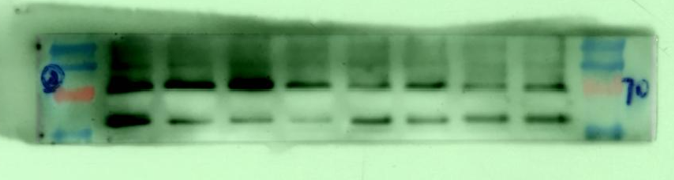  chemi: 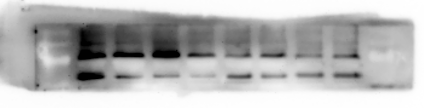 |  |  |
|  | **Ncoa4**  color+chemi: 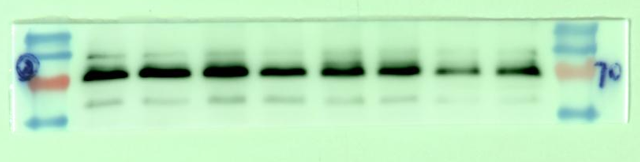  chemi: 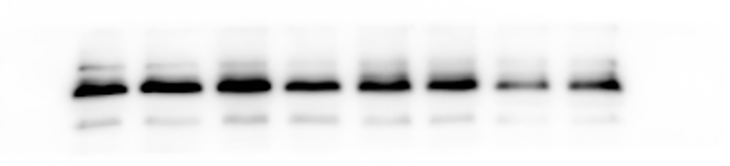 |  |  |
|  | **Fth**  color+chemi: 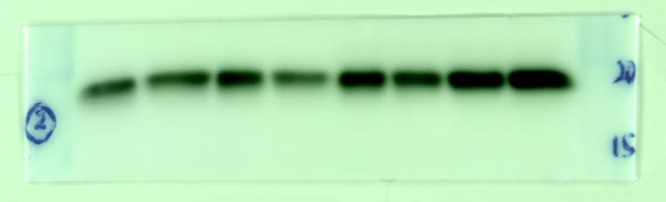  chemi: 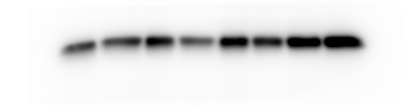 |  |  |
|  | **Ftl**  color+chemi: 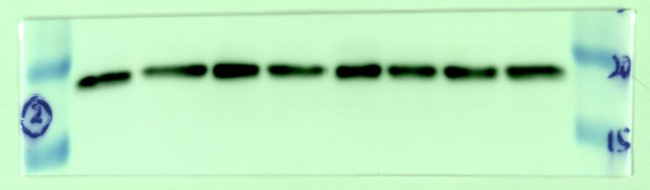  chemi: 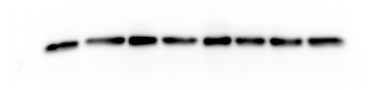 | 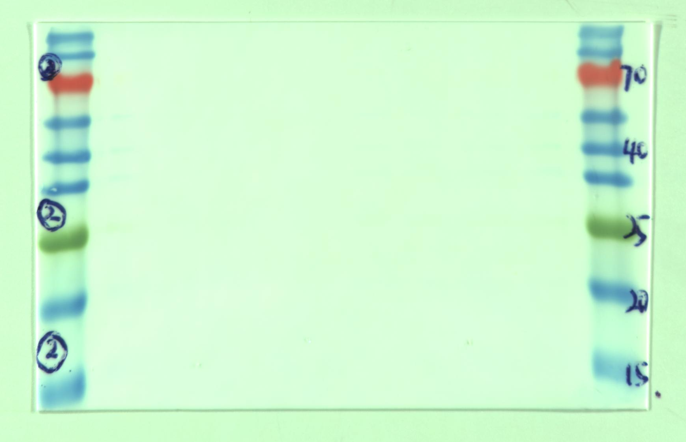 | 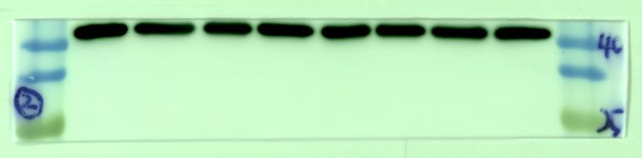 |
|  | **Ctsk**  color+chemi: 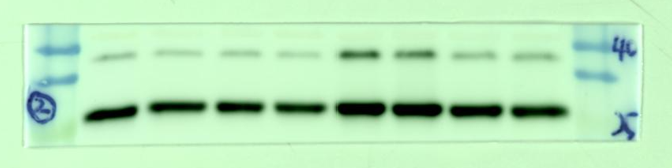  chemi: 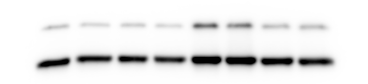 |  |  |
|  | **Nfatc1**  color+chemi: 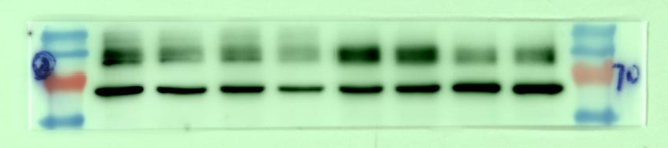  chemi: 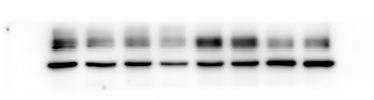 |  |  |
|  | **β-Actin**  color+chemi: 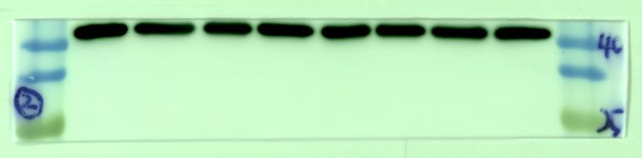 chemi: 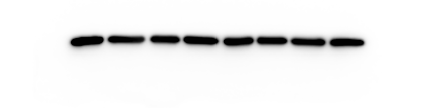 |  |  |

| Image in figure | Original image | Original full image  (with same handwritten number) | β-Actin of original full image |
| --- | --- | --- | --- |
| Fig. 5C left  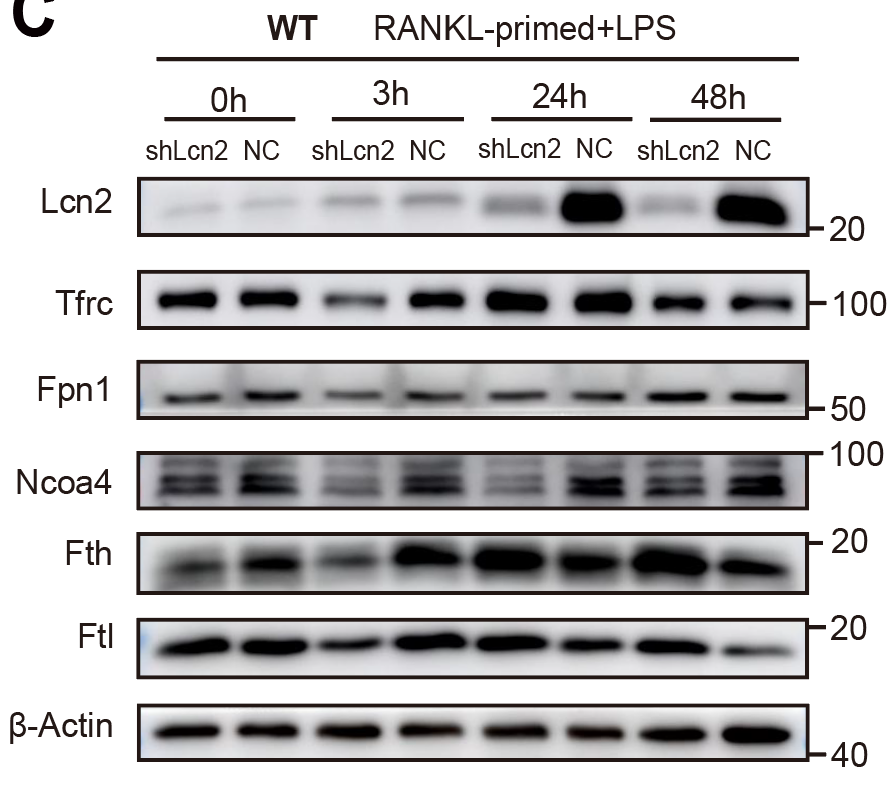 | Lcn2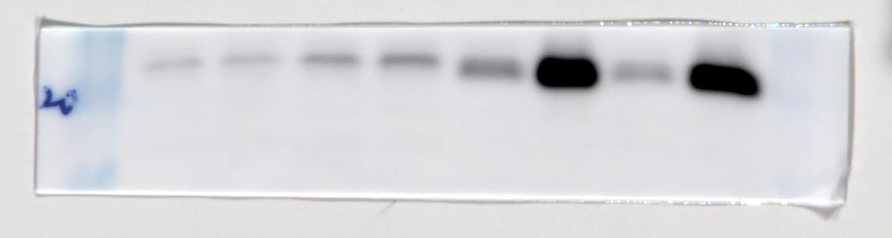 | 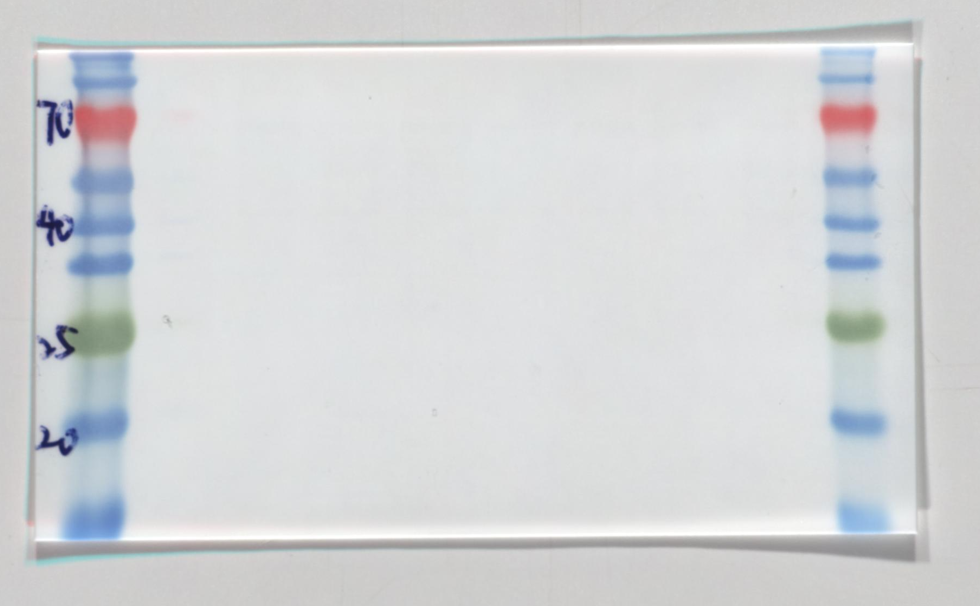 | 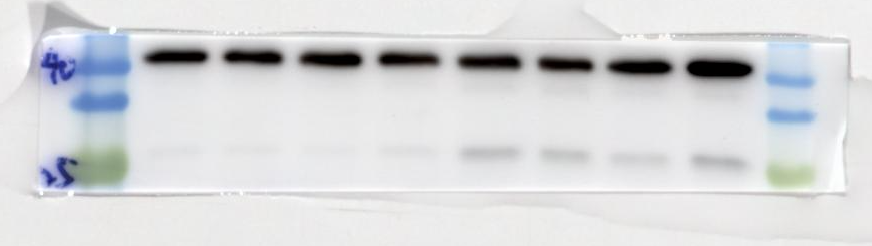 |
|  | Fpn1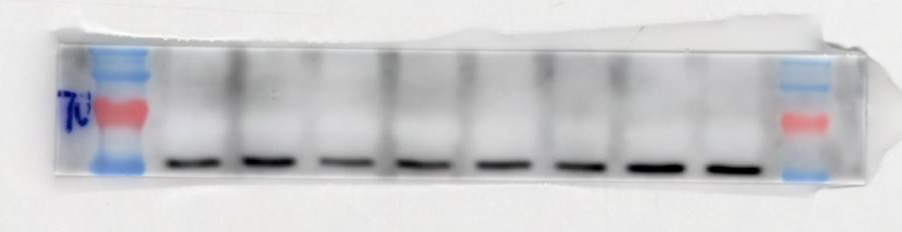 |  |  |
|  | Ncoa4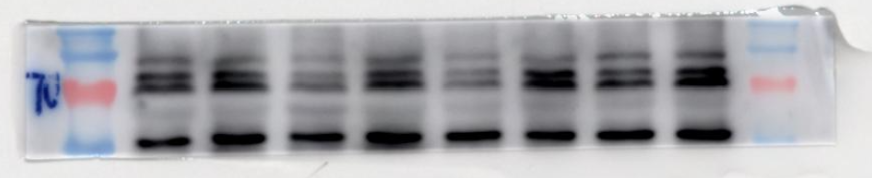 |  |  |
|  | β-Actin  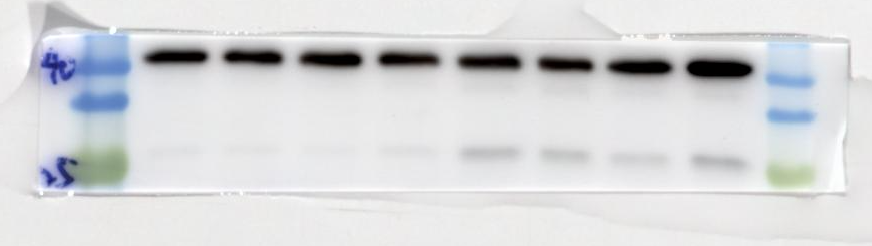 |  |  |
|  | Fth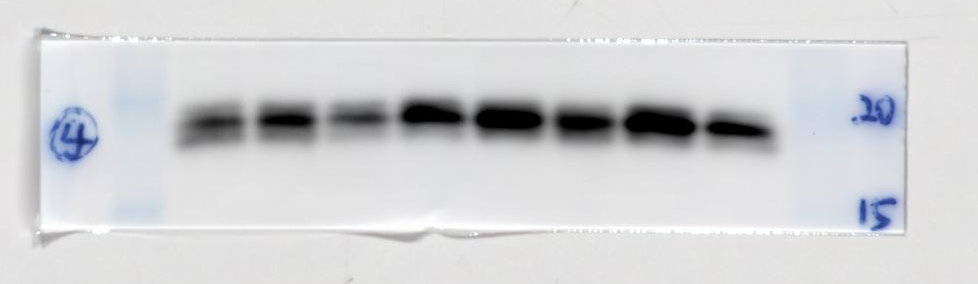 | 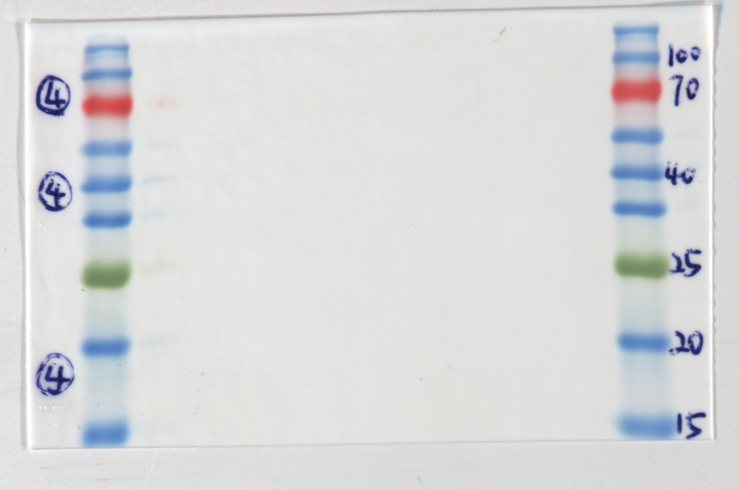 | 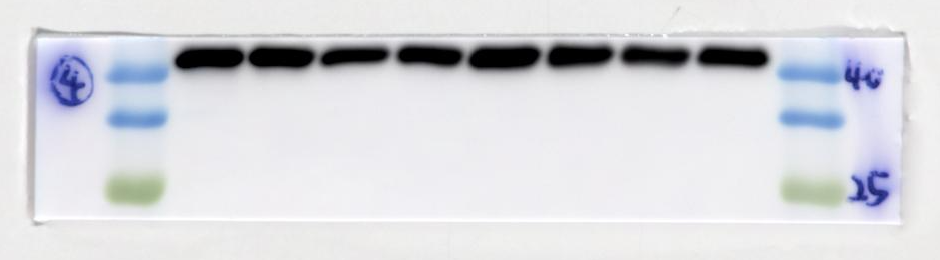 |
|  | Ftl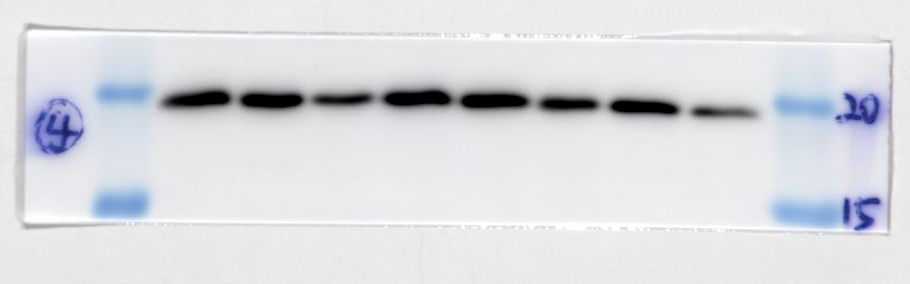 |  |  |
|  | Tfrc 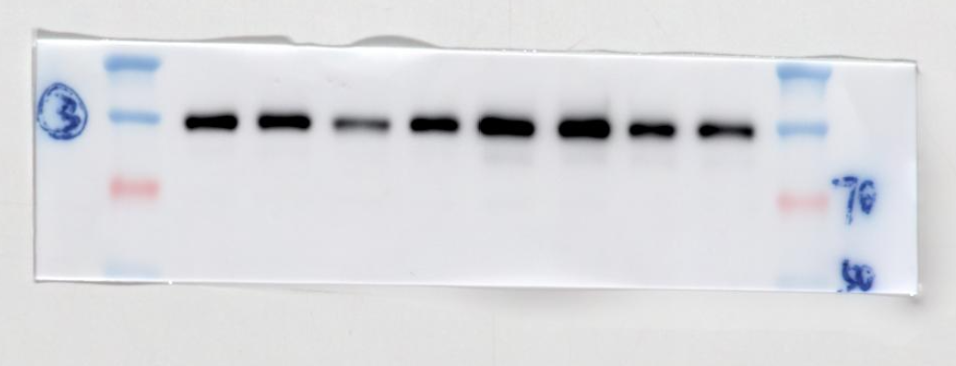 | 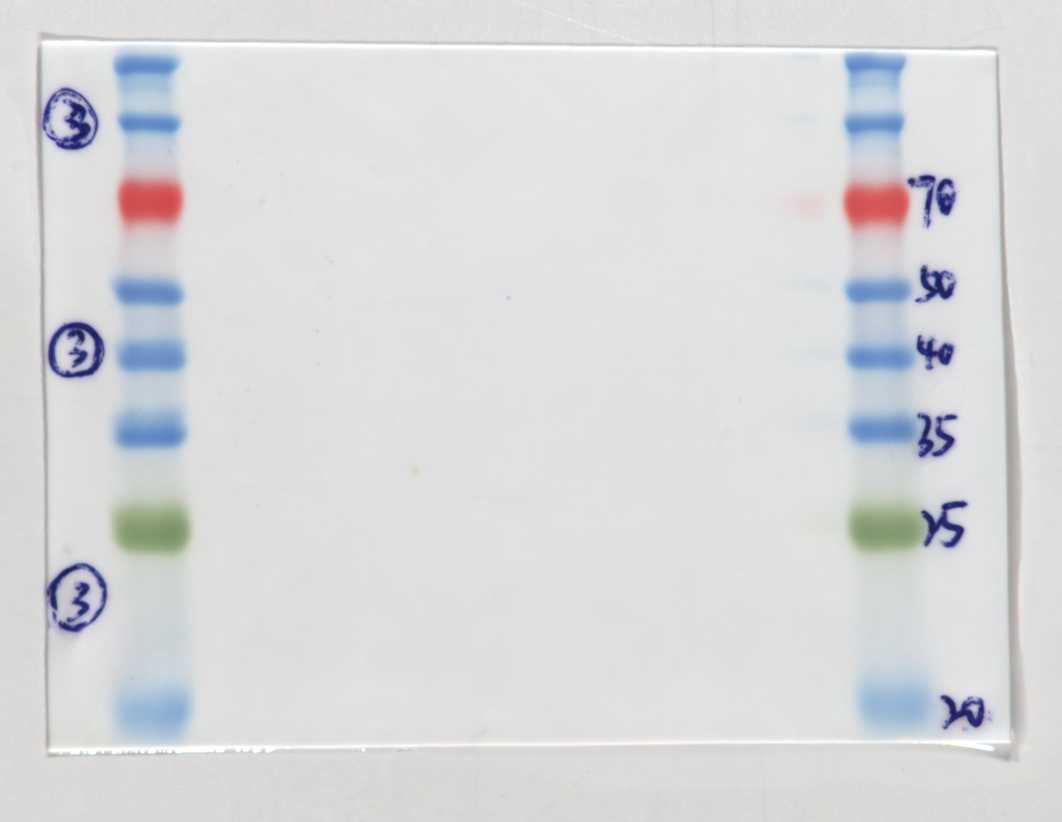 | 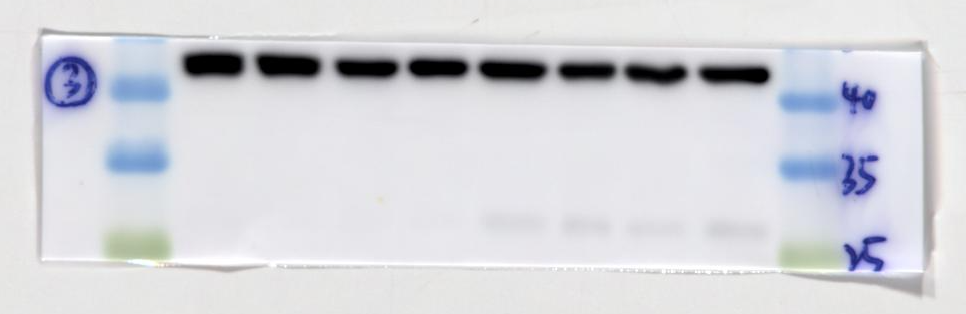 |

| Image in figure | Original image | Original full image  (with same handwritten number) | β-Actin of original full image |
| --- | --- | --- | --- |
| Fig. 5C right  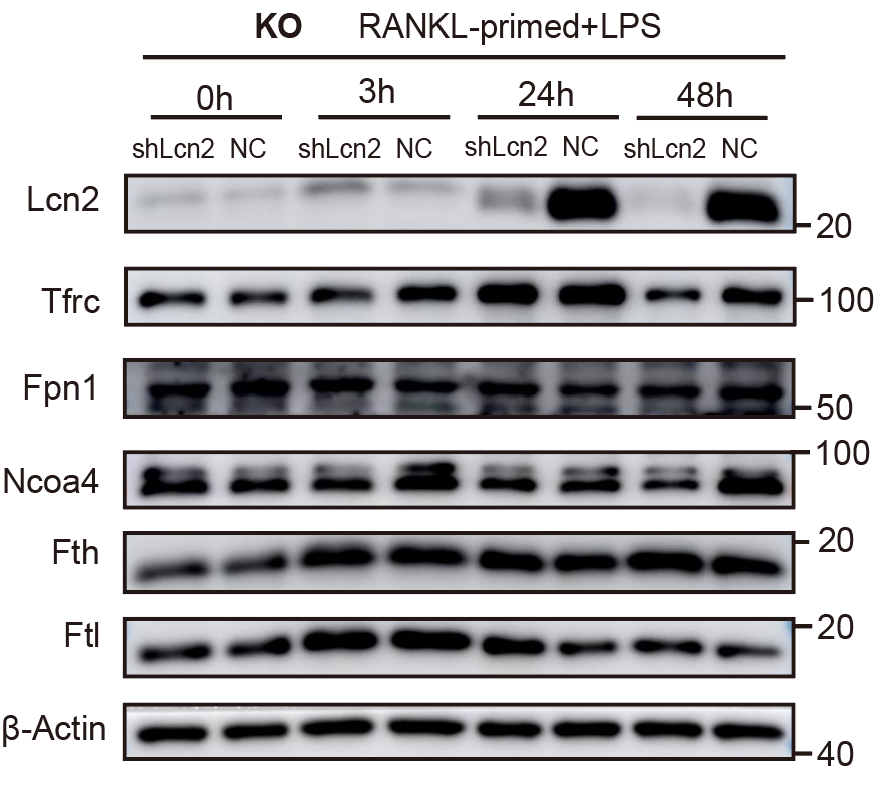 | Lcn2 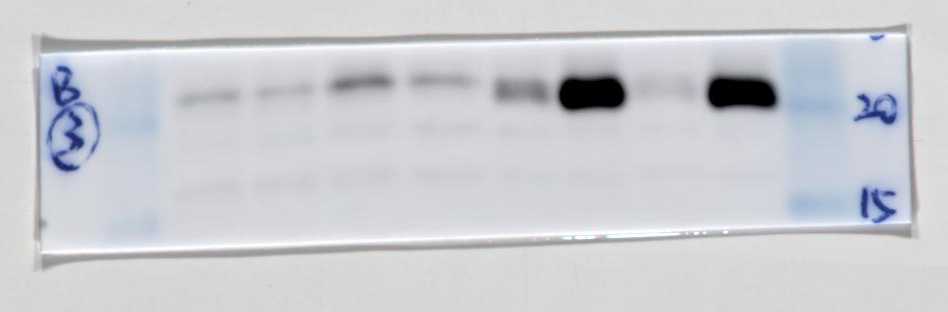 | 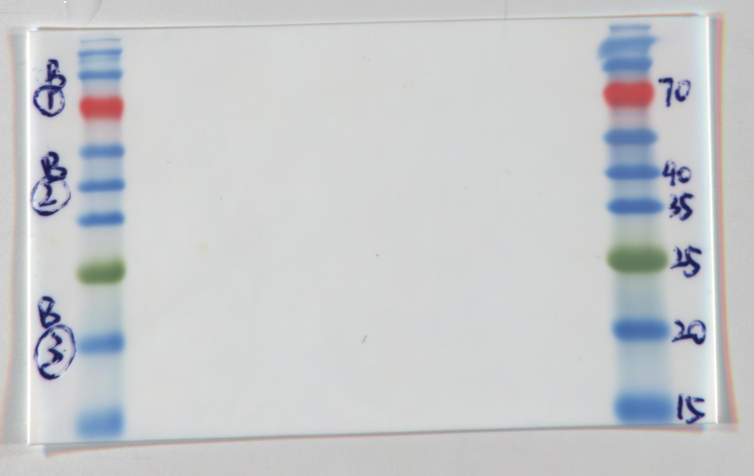 | 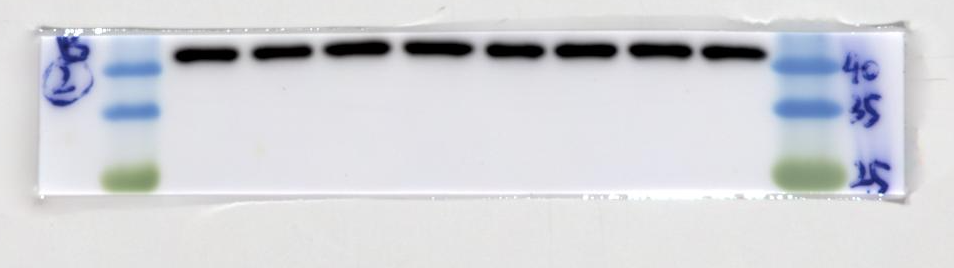 |
|  | Tfrc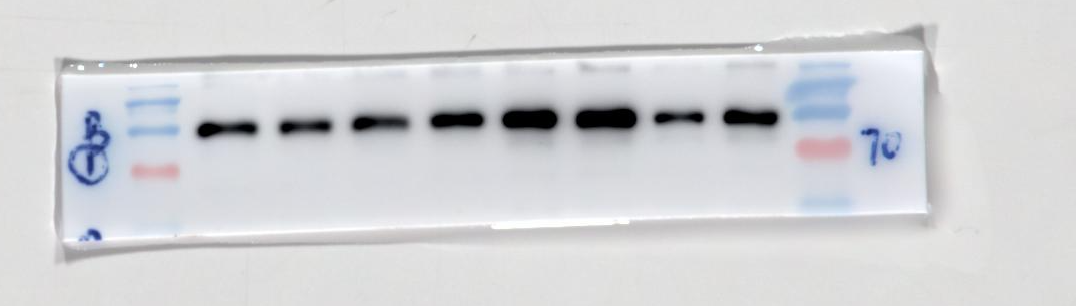 |  |  |
|  | Fpn1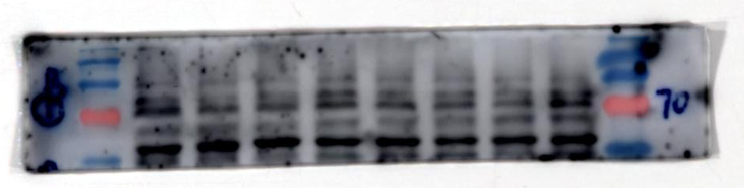 |  |  |
|  | Ncoa4 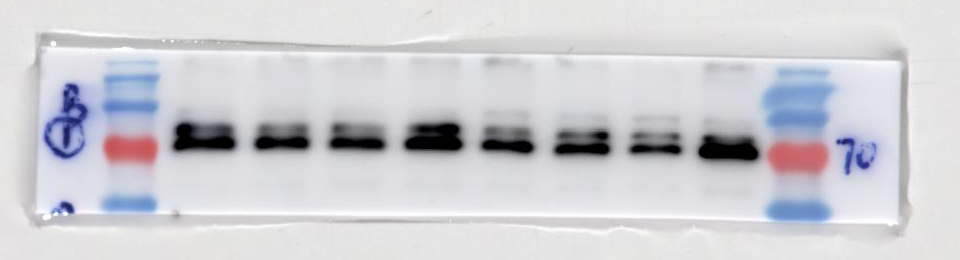 |  |  |
|  | Fth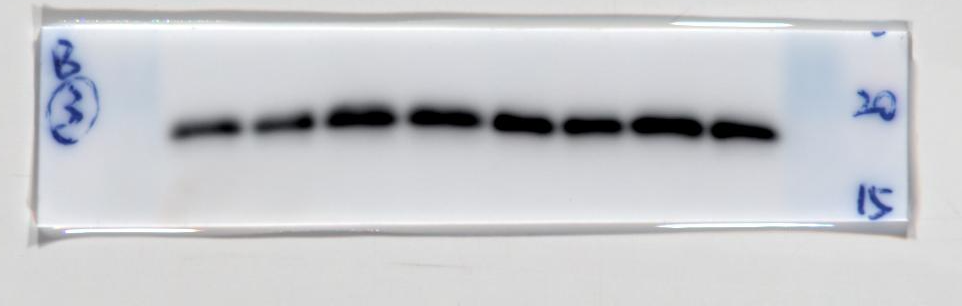 |  |  |
|  | Ftl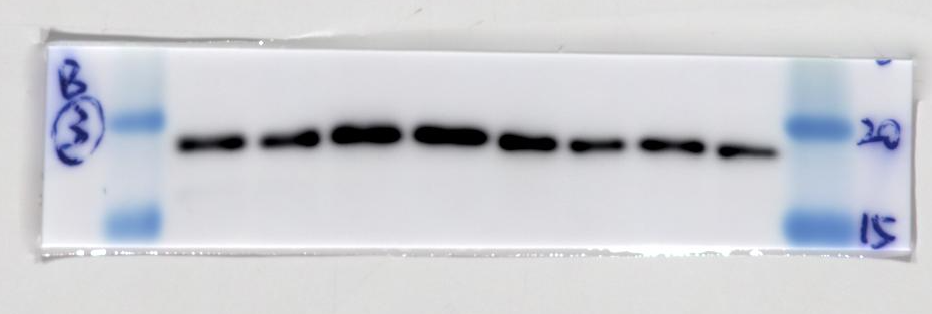 |  |  |
|  | β-Actin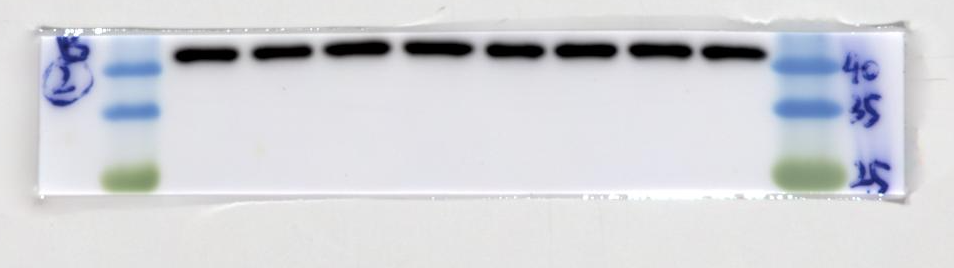 |  |  |

| Image in figure | Original image | Original full image  (with same handwritten number) | β-Actin of original full image |
| --- | --- | --- | --- |
| Fig. S2D up  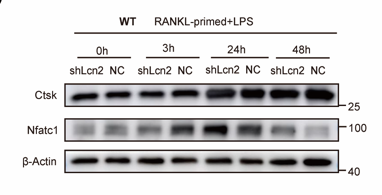 | Ctsk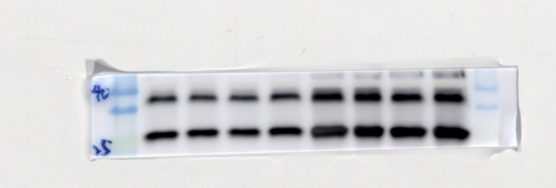 | 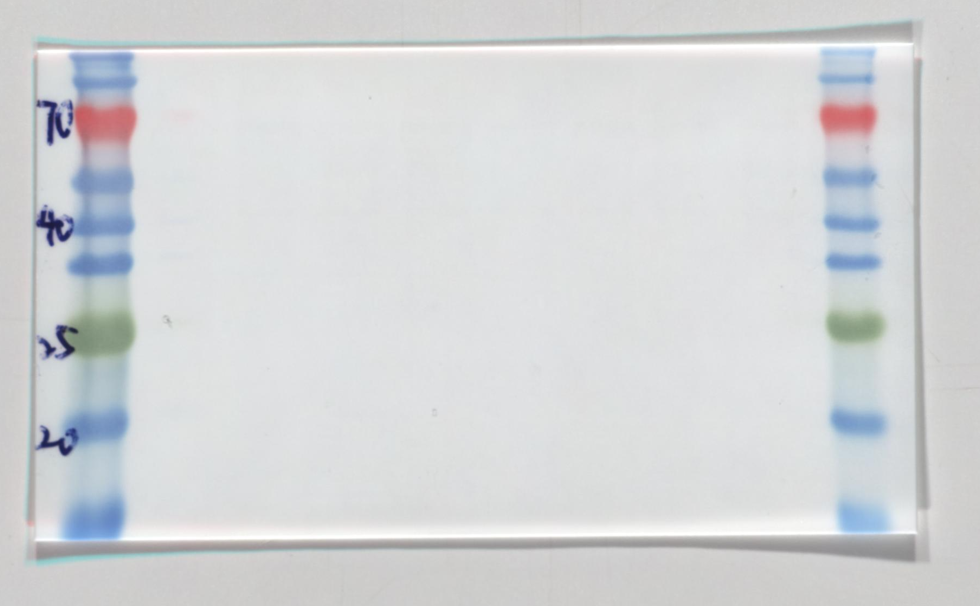 | 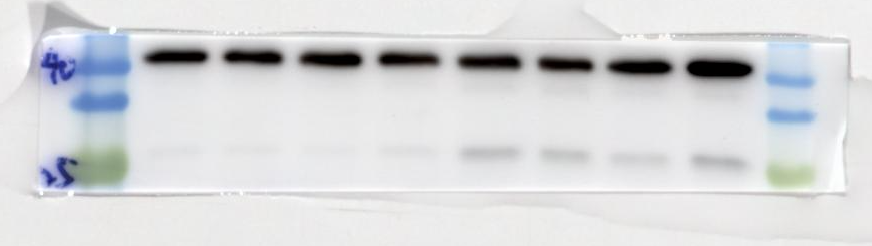 |
|  | β-Actin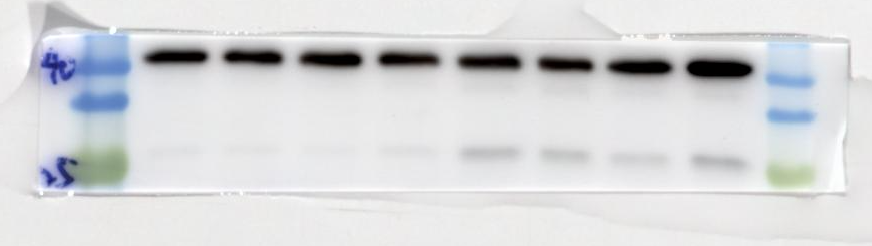 |  |  |
|  | Nfatc1  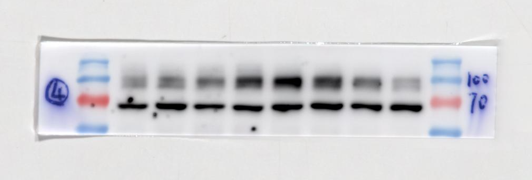 | 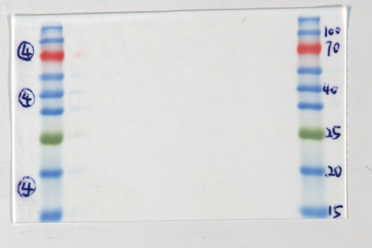 | 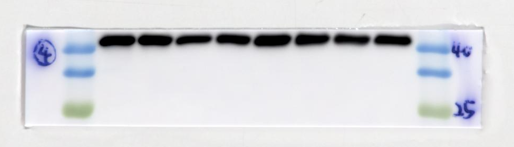 |
| Fig. S2D down  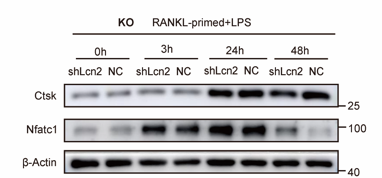 | Ctsk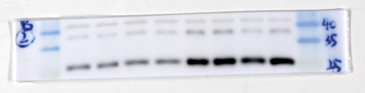 | 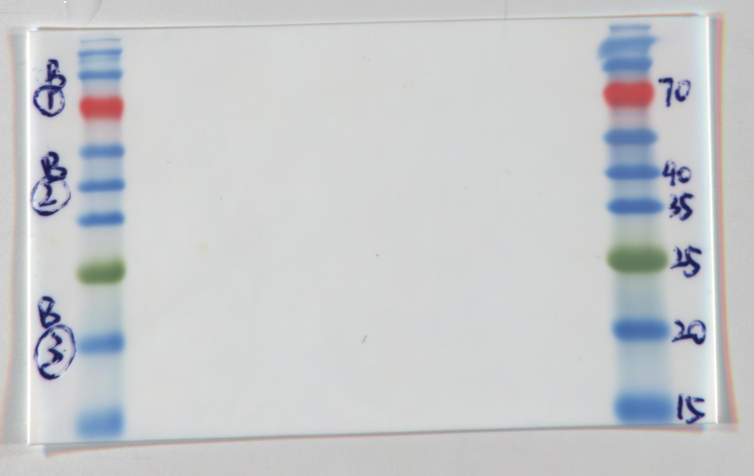 | 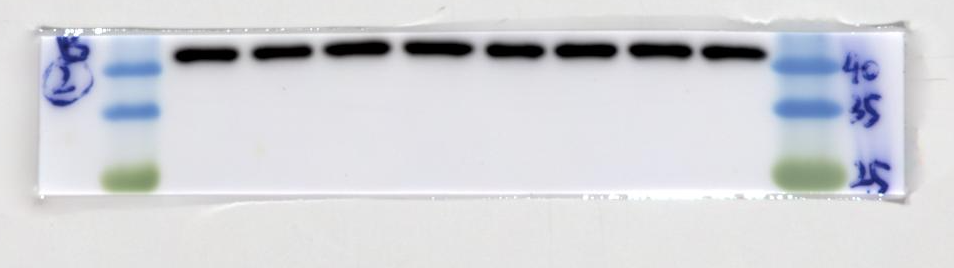 |
|  | Nfatc1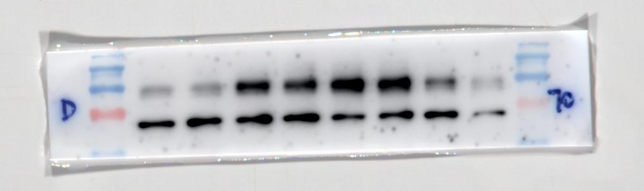 | 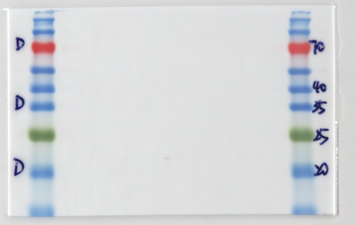 | 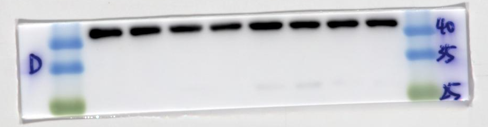 |
|  | β-Actin  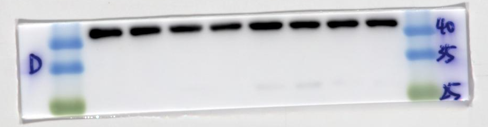 |  |  |
